# Transcriptome resilience predicts thermotolerance in *Caenorhabditis elegans*

**DOI:** 10.1186/s12915-019-0725-6

**Published:** 2019-12-10

**Authors:** Katharina Jovic, Jacopo Grilli, Mark G. Sterken, Basten L. Snoek, Joost A. G. Riksen, Stefano Allesina, Jan E. Kammenga

**Affiliations:** 1 Laboratory of Nematology, Wageningen University, Droevendaalsesteeg 1, Wageningen, 6708 PB The Netherlands; 2 Department of Ecology and Evolution, University of Chicago, 1101 E 57th St, Chicago, IL 60637 USA; 3 Santa Fe Institute, 1399 Hyde Park Rd, Santa Fe, NM 87501 USA; 4 The Abdus Salam International Center for Theoretical Physics (ICTP), Strada Costiera 11, I-34014 Trieste, Italy; 5 Theoretical Biology and Bioinformatics, Utrecht University, Padualaan 8, Utrecht, 3584 CH The Netherlands

**Keywords:** Heat stress, Recovery, *C. elegans*, Resilience, Thermotolerance, Gene expression dynamics, Transcriptome

## Abstract

**Background:**

The detrimental effects of a short bout of stress can persist and potentially turn lethal, long after the return to normal conditions. Thermotolerance, which is the capacity of an organism to withstand relatively extreme temperatures, is influenced by the response during stress exposure, as well as the recovery process afterwards. While heat-shock response mechanisms have been studied intensively, predicting thermal tolerance remains a challenge.

**Results:**

Here, we use the nematode *Caenorhabditis elegans* to measure transcriptional resilience to heat stress and predict thermotolerance. Using principal component analysis in combination with genome-wide gene expression profiles collected in three high-resolution time series during control, heat stress, and recovery conditions, we infer a quantitative scale capturing the extent of stress-induced transcriptome dynamics in a single value. This scale provides a basis for evaluating transcriptome resilience, defined here as the ability to depart from stress-expression dynamics during recovery. Independent replication across multiple highly divergent genotypes reveals that the transcriptional resilience parameter measured after a spike in temperature is quantitatively linked to long-term survival after heat stress.

**Conclusion:**

Our findings imply that thermotolerance is an intrinsic property that pre-determines long-term outcome of stress and can be predicted by the transcriptional resilience parameter. Inferring the transcriptional resilience parameters of higher organisms could aid in evaluating rehabilitation strategies after stresses such as disease and trauma.

## Background

Temperature is a key factor that directly affects physiological processes, life history, and behavior of many organisms. Ambient temperatures can rise suddenly, inflicting physiological consequences often lasting far beyond the initial exposure. For instance, it has repeatedly been shown that exposure to heat stress early in life can have an effect on traits later in life such as reproductive success and lifespan in the nematode *Caenorhabditis elegans* and fruit fly *Drosophila melanogaster* [1–5]. The ability to withstand the negative effects of heat stress is called thermotolerance and requires instant regulatory protective responses involving the well-studied heat-shock response [6]. Since tolerance is a trait that results in the absence of adverse effects, it is difficult to predict tolerance levels of an organism before the negative effects of stress have become apparent.

Next to the induction of genes within specific stress response pathways, recent studies in *C. elegans* have shown that heat stress also induces a broad acclimation of transcriptional patterns involving differential expression of thousands of genes [7–9]. Furthermore, during prolonged stress exposure, expression changed continuously until lethal stress levels were reached [8]. Those findings illustrate that the state of the transcriptome directly reflects the stress levels the organism was exposed to. While the reactive processes occurring during the heat-shock response are well understood, much less is clear about how organisms recover from a heat shock and how the genome-wide transcriptional state might be used to predict long-term outcome of a short bout of heat stress.

Here, we quantify gene expression resilience during and after heat stress in order to predict thermotolerance in *C. elegans.* First, by measuring genome-wide gene expression levels of the canonical laboratory strain Bristol N2 in three high-resolution time series (development, heat stress, and recovery from heat stress), and applying a principal component analysis to the data, we show that the state of the transcriptome during and after the dynamic response to heat-stress perturbations can be captured by a single parameter. This finding provides the basis to evaluate and compare complex transcriptional patterns after stress in a straightforward and quantitative way.

Secondly, in order to generalize our findings beyond the individual genotype, we expanded our analyses across different genetic backgrounds. Previous research shows that different genotypes are differently affected by the heat stress [3, 9, 10], assumingly due to an intrinsic difference in thermal tolerance. Our results show that transcriptome resilience measured after a mild heat stress early in the development of *C. elegans* is predictive of its thermotolerance. Thermotolerance (based on long-term survival) and transcriptional resilience were measured in independent populations of the same genotype, emphasizing the genetically intrinsic nature of thermal tolerance and the robustness of this approach to predict thermal tolerance. Our methods are straightforward to implement and allow to map gene expression data during and after heat stress onto a few main quantitative scales that have a clear biological interpretation.

## Results and discussion

### Using principal component analysis to infer a developmental axis *D* and heat-stress axis *H*

**Fig. 1:**
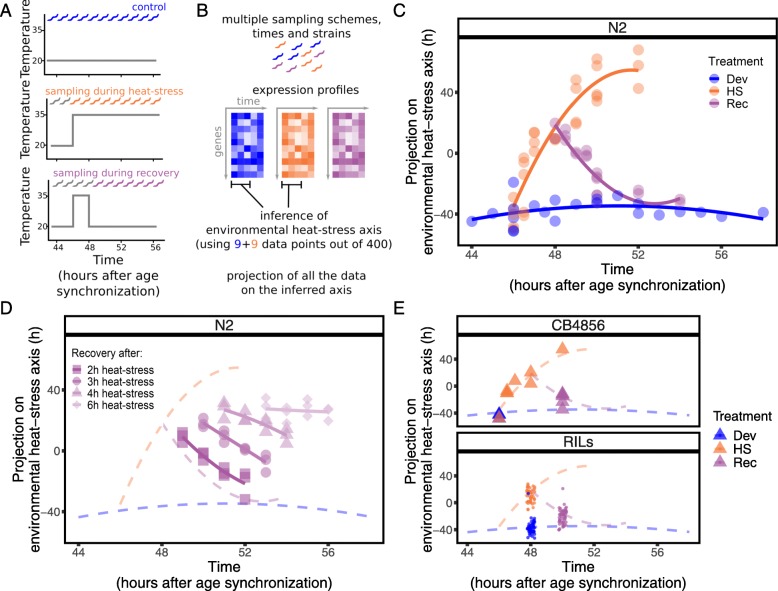
Experimental setup and expression dynamics during heat-stress perturbations. **a** Experimental design of the three main treatments: control (blue; 20 °C throughout development), heat stress (orange; populations shifted to 35 °C after 46 h of development at 20 °C), and recovery (purple; at 20 °C after heat stress). **b** A subset of samples from heat stress and control treatments was used for the inference of the heat-stress axis, *H*, describing the gene expression dynamics during heat stress. **c** Projection of the data on this axis describes the dynamics of the response to heat stress. Notably, this is true also for the recovery data that was not used to infer axis *H*. **d** Projection of transcriptome data of the recovery process after 2, 3, 4, and 6 h of heat stress shows a decrease in recovery dynamics. **e** Axis *H* also describes the transcriptional heat-stress response for strains other than N2

*C. elegans* develops relatively fast—within ~ 65 h an individual develops from an egg into a reproducing adult [11]. The transition through the four larval stages is controlled by highly dynamic transcriptional processes [12, 13]. To characterize the temporal dynamics in genome-wide gene expression during heat stress and in recovering *C. elegans* populations, we have to remove stress-independent variation in gene expression patterns caused by differences in development between samples collected in a time series spanning several hours. For this purpose, we compiled a data set of 71 gene expression profiles measured in isogenic populations of the canonical strain Bristol (N2) sampled in an approximately hourly interval during exposure to three different treatments: (i) during unperturbed development at 20 °C [12], (ii) during prolonged exposure to heat-stress conditions at 35 °C [8], or (iii) during a period of recovery at 20 °C after a 2-h heat stress at 35 °C (Fig. 1a). First, the data was separated into training and testing sets (as indicated in Additional file 1: Table S1). Second, through the application of principal component analyses on expression profiles from unperturbed populations (*n* = 9 samples out of 18) and from heat-stressed populations (*n* = 9 samples out of a total 39), we inferred the combination of gene expression patterns that best characterized the overall expression dynamics of each treatment (Fig. 1b; for background information on principal components see Additional file 2).

For the unperturbed data, we found that the second principal component shows a clear trend with the developmental age of the samples (Additional file 2: Figure S3). Therefore, while also the third and fourth principal components capture time-dependent expression changes (Additional file 2: Figure S2C), the second principal component is sufficient to capture the transcriptional age of the age-synchronized N2 populations used. From here on, the second principal component obtained from the unperturbed data is named *developmental axis D*, describing the temporal expression dynamics during development.

Subsequently, the developmental influences captured by *D* were removed from the data set of heat-stressed nematodes, allowing for the inference of the *heat-stress axis H*. *H* describes temporal expression patterns induced by heat stress, while disregarding heat-stress-independent temporal transcriptional patterns. Hence, by combining the data of perturbed and unperturbed populations, we were able to disentangle the effects of development and heat stress in time.

### Heat-stress axis *H* reflects exposure duration, as well as recovery from heat stress

By projecting gene expression profiles on the heat-stress axis *H*, each sample can be associated with a value *h.* While only 18 samples were used to infer the axis, all 71 samples from all three time-series align along the axis according to treatment and exposure duration (Fig. 1c, and Additional file 2: Figure S5), showing that *h* is a quantitative measure of the transcriptional stress response. The value of *h* increased with increasing heat-stress duration (Fig. 1c, orange) until *h* started to saturate after long exposure (> 4 h). The unperturbed worms had a constant value of *h* (Fig. 1c, blue), showing that we were able to successfully remove the signal caused by developmental differences on gene expression. Strikingly, even though samples collected during recovery were not used to determine the axis *H*, the gene expression during recovery from a 2-h heat stress was also well-explained with samples returning to the level of *h* typical of unperturbed worms within about 4 h (Fig. 1c, purple). We concluded that *h* quantitatively reflects exposure duration, as well as the time elapsed since the end of exposure. Note that although samples returned to the pre-stress treatment level of *h* after recovery, this does not imply that recovered *C. elegans* populations are transcriptionally indistinguishable from unperturbed ones (as, for instance, can be seen by projecting recovery samples onto *D*, and their failure to return to the pattern of unperturbed development see Additional file 2: Figure S3B). Therefore, in this context, recovery was defined and measured here by the ability to depart from stress response dynamics.

So far, the results have shown that the transcriptional recovery process after a mild stress can be followed over time using the heat-shock axis *H*. To exclude the possibility that *H* only captured time since the end of the heat stress without biological meaning towards phenotypic recovery or resilience, we expanded the dataset to include four additional time-series tracking the transcriptome recovery for 4 h following four different heat-stress intensities (2, 3, 4, and 6 h at 35 °C). The long-term effects of these stress intensities on survival, reproduction, and mobility have been shown to range from mild after short (2 h) exposure to 100% mortality within 24 h after 6 h at 35 °C [8]. Figure 1 d shows that mildly stressed population transcriptionally returned to pre-stress levels of *h* during the observed recovery period, while increasing stress duration led to a slowing down of the transcriptional recovery process, and severely stressed populations remained at a constant high value of *h.* Therefore, *H* can distinct between the progress of the recovering transcriptome and a non-recovering transcriptome.

### Heat-stress axis *H* retains essential features of the biology of the heat-stress response

**Fig. 2:**
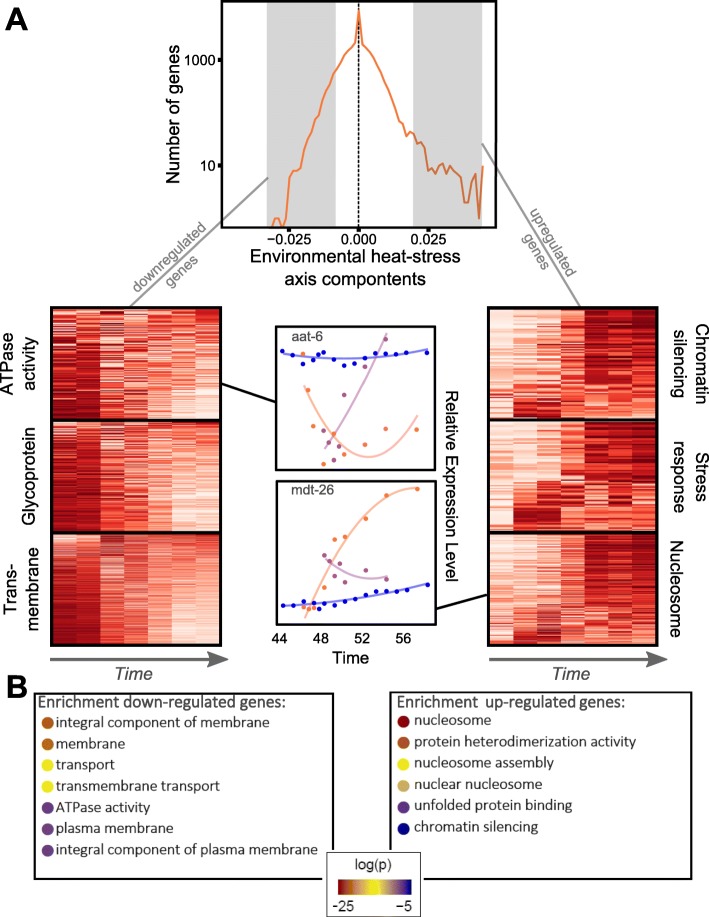
Single gene contribution to heat-stress axis, *H*. **a** Distribution of the entries of the heat-stress axis (top of the figure). The distribution is not symmetric, which means that more genes contributing to this axis (relatively to their unperturbed level of expression) are upregulated as a response to heat stress. Examples of gene expression dynamics of selected GO terms during heat stress shown in heat-maps, as well as two examples of genes with negative and positive components (gene expression measured during heat stress in orange, during recovery in purple, and blue corresponds to development). **b** Results of an enrichment analysis performed with DAVID 6.8 of the top 5% of genes with the highest contribution to *H.* Shown here are GO terms with a Bonferroni-corrected *p* value below 0.05

Having shown that the axis *H* recapitulates the transcriptional state during and after exposure to heat stress, we investigated the biological properties of the axis *H*. To this end, we performed an enrichment analysis to determine which groups of genes contributed the most to the axis *H* (Fig. 2; full enrichment output in Additional file 1: Table S3). Consistent with expectations, genes encoding for stress response proteins (in particular heat-shock proteins *hsp*) and nucleosomes (in particular histones *his*) were upregulated. These gene classes have previously been shown to be highly activated by heat stress [7–9, 14]. Similarly, the value of *h* is negatively correlated with some genes involved in cell metabolism such as ATPase transmembrane proteins. This analysis shows that the axis *H* retains essential features of the biology of the heat-stress response, while summarizing these complex biological dynamics into a single quantitative parameter.

### Heat-stress axis *H* reflects the average heat-stress response across multiple genotypes

Next, we tested whether heat-stress axis *H* can also reflect the change in gene expression for different genotypes. We used expression profiles of the strain CB4856 (Hawaii), which is genetically distinct from N2, as well as 54 recombinant inbred lines (RILs) [9], which are genetic mosaics derived from a cross between CB4856 and N2 [15, 16]. Gene expression in these different genotypes was measured in control conditions, during heat stress, and in recovery. Projections of the expression profiles onto *H* followed a similar pattern as N2 (Fig. 1e). To further analyze the variation in projections among genotypes, we compared the standard deviation of the projection in N2 replicas with the variation found between RILs (this comprised computing the distribution of SDs of the projection of 5000 randomly selected subsets of RIL data for each separate treatment of the same sample size as the corresponding N2 data; see Additional file 2: Section S3 and Fig. S7). We found that the heat-stress axis *H* does not resolve genotype-dependent differences in the response to stress, but rather successfully recapitulates the *average* dynamic transcriptional response of these different genotypes. The robustness of the pattern across genotypes reflects the high degree of conservation in transcriptional resilience.

A common use of the CB4856 x N2 recombinant populations is the genetic mapping of traits to determine which genetic regions are responsible for trait variation, a method called quantitative trait loci (QTL) mapping (for more information, see [17, 18]). It should be noted that, in the present study, the RILs were not used for the genetic mapping of traits, but rather as a genotypic library. To link our findings to genetic mapping of gene expression conducted previously in N2 x CB4856 RIL populations at various ages and in different treatments [9, 15, 19–22], we performed an enrichment analysis of genes contributing to *H.* There is a strong enrichment of *H* in *cis*- and *trans*-eQTL mapped in the RIL heat-shock experiment of Snoek and Sterken [9] (Additional file 3: Figure S10). This indicates that genetic variation in RIL panels affects expression of genes that strongly contribute to *H* (also supported by enrichment analysis on polymorphic genes; Additional file 3: Figure S11). Interestingly, the upregulated genes in *H* are enriched for in 7 out of 11 (cis-)eQTL experiments, suggesting that genetic variation in N2 x CB4856 populations is linked to differences in stress response.

### Variation in stress resilience across genotypes is captured in a genetic heat-stress axis (*GH)*

We have shown that the heat-stress axis *H*, inferred using solely the isogenic strain N2, describes the *average* conserved stress response of a library of highly divergent genotypes. On the other hand, there is large natural variation in long-term effects of heat-stress exposure across genotypes, for example marked by differences in the stressed transcriptome [9], survival rates [3, 23], and reproductive rates [3]. Considering that trait variation is genotype dependent, it implies a difference in transcriptional resilience during and/or after stress. Next, we ask if a single axis could also capture the natural variation in heat-stress response across genotypes. Since genotypes differ in more traits than their transcriptional response to stress, such as developmental timing and size, we needed to isolate stress-induced variation in expression levels from other intrinsic differences in the transcriptome between genotypes. For this purpose, we used gene expression data of RILs collected before and after 2 h of heat stress [9]. Analogous to our approach above in inferring the heat-stress axis *H* for N2 by removing developmental differences, we corrected the heat-stress response of the RILs for their intrinsic gene expression differences in unperturbed conditions (see Additional file 2: Section S3). We inferred a *genetic heat-stress axis* (*GH*) that isolates and describes the variability across strains in their stress response.

To substantiate this observation, we conducted an enrichment analysis of genes strongly contributing to *GH* (Additional file 1: Tables S2-S4) and found a strong enrichment for *trans*-eQTL in heat-shock induced eQTL [9] (Additional file 3: Figure S10). This indicates that *GH* captures multiple genes of which the expression was affected by natural variation. In support, analysis of polymorphisms in the *GH* genes revealed that these genes often had polymorphisms in the 5′ regulatory region (Additional file 3: Figure S11). In conclusion, we propose that *GH* is a summary of the *trans*-eQTL architecture.

**Fig. 3:**
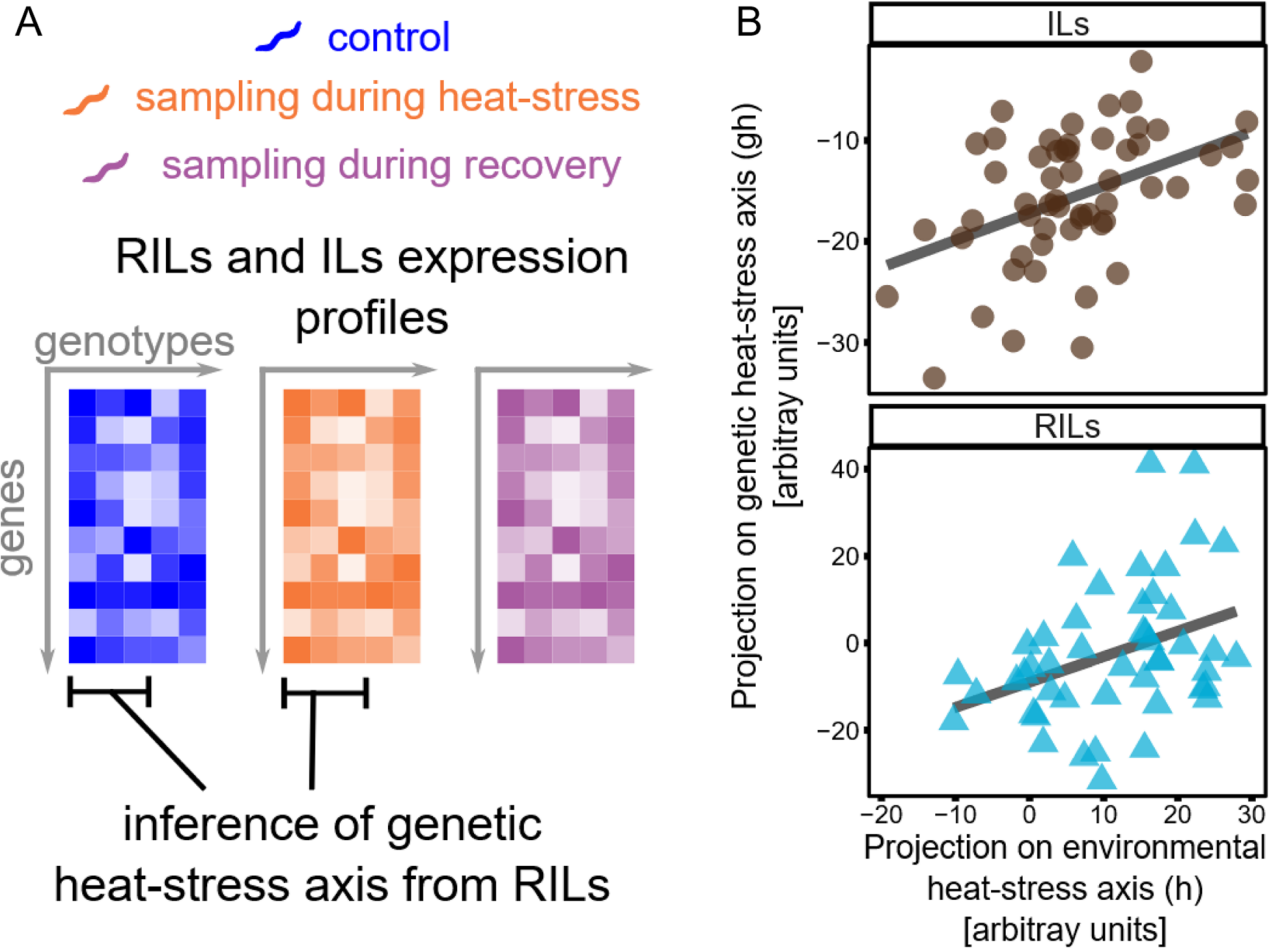
Derivation of the genetic heat-stress axis, *GH*, and relation with environmental heat-stress axis, *H.* **a** For each RIL and IL, we measured gene expression in absence of perturbation, after 2 h exposure to heat stress and during recovery (after 2 h of the end of a 2-h heat stress). Using only RIL data, we obtained the genetic heat-stress axis (*GH*), describing the difference between RILs in heat-stress response (discounting their differences in gene expression in the unperturbed case). **b** Correlation between genetic heat-stress axis and the environmental heat-stress axis shown for heat-stress samples of RILs and ILs

The strength of relationship between the genetic axis *GH* and the environmental heat-stress axis *H* measures the proportion of the variation of heat-stress response across RILs that is due to timing differences. We found a positive correlation between the two axes (Spearman rho = 0.36, *p* = 0.01) implying that different strains respond as if they were exposed to the heat stress for different durations. This was confirmed by analyzing a second set of heat-stressed gene expression profiles from a separate alternative panel of inbred lines [24, 25] (Introgression Lines, ILs; Spearman rho = 0.44, *p* = 8 × 10^− 4^) (Fig. 3). These results show that the genetic differences also lead to difference in the timing or magnitude of the transcriptional response. The presence of a correlation between the axes *H* and *GH* also implies that the value of *gh* (the projection of the gene expression profile on the axis *GH*) recapitulates the relative strength of the heat-stress response: the higher the value of *gh*, the farther away is the gene expression profile from the unperturbed state or, in other words, the lower its transcriptional resilience to heat stress.

### Transcriptional resilience on a short timescale is predictive of the variability in thermotolerance on a longer timescale

**Fig. 4:**
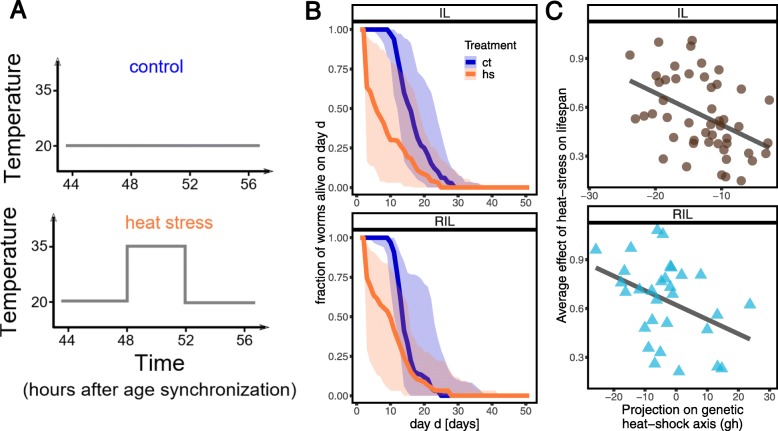
Effect of heat stress on lifespan and correlation with gene expression recovery. **a** Experimental setup used to collect lifespan data of 40 RILs and 54 ILs. An average of 31 animals were scored per genotype and treatment combination. **b** Comparison of the cumulative lifespan distribution of unperturbed (blue) and perturbed (orange) RILs and ILs. Thick lines correspond to the average, while the shaded area displays the 95% confidence interval. **c** Effect of heat stress on lifespan (measured as the ratio of the average lifespan after perturbation and without perturbation) correlates with the projection of recovery data on the genetic heat-stress axis for RILs and ILs. Strains recovering faster from heat stress experience a weaker effect on their lifespan

Heat stress affects gene expression dynamics and resilience in the short term in a predictable way, which is recapitulated by axes *H* and *GH*. On the other hand, in the long run, heat stress also affects developmental speed, aging, behavior, and vitality—for instance by drastically reducing lifespan. We set out to explore how the variability in gene expression dynamics following heat stress on a short timescale is predictive of variability in thermotolerance measured on a longer timescale. Thermotolerance in *C. elegans* can be recorded by its survival rates. Therefore, in a parallel experiment, we collected lifespan data of over 200 different RILs and ILs with and without exposure to heat stress. While 2 h at 35 °C are sufficient to induce a strong transcriptional response, previous experiments have shown that overall lifespan is not necessarily shortened at this intensity [8]. Therefore, we increased the exposure duration to 4 h at 35 °C for lifespan measurements as this duration is known to affect lifespan [3, 8], allowing us to make a better estimate of difference in thermotolerance across genotypes. As expected, both RILs and ILs show high variability in their lifespan after heat stress and in control conditions. On average, the lifespan following a heat stress was lower than what was found for unperturbed populations (Fig. 4; logrank *p* < 0.001 for RILs as well as ILs). When comparing individual genotypes, 53 lines were significantly affected by heat stress (logrank, FDR < 0.05), while 28 lines were not strongly affected (i.e., they displayed a higher thermotolerance; for lifespan curves of the individual lines see Additional file 4, and for the full statistical output see Additional file 1: Table S6).

Next, we compared the effect of heat stress on the lifespan of different RILs with the difference in transcriptional resilience, measured by projecting the recovery data of the RILs on the genetic heat-stress axis *GH*. Figure 4 shows that the ability of different strains to recover from heat stress is predictive of thermotolerance (Spearman rho = − 0.41, *p* = 0.02; Fig. 4). In order to test the robustness of this result, we also performed the same analysis on ILs, which are genetically mostly derived from one strain (N2) and were not used to infer the axis. In this case, we also found a significant correlation (Spearman rho = − 0.46, *p* = 10^− 3^), implying that the connection between the ability to recover and lifespan was robust across different inbred line panels. The projection of the heat-stress data onto *GH* (which is related to the speed at which worms react to heat stress) was not robustly correlated with lifespan (see Additional file 3: S12-S15), showing that resilience measured based on recovery data was more directly linked to tolerance.

## Conclusions

This study sheds light on how organisms recover from environmental stress perturbations, by means of a systemic *modus operandi* based on using genome-wide gene expression profiles. We conclude that a relatively simple axis can measure stress resilience of a dynamic transcriptome in a single quantitative variable and describes the capacity of an organism to recover from heat stress. Our findings show that natural variation in transcriptome resilience after mild stress exposure is predictive of thermotolerance across a diverse set of genotypes in *C. elegans*. The results imply that thermotolerance is an intrinsic trait that largely pre-determines long-term effects of heat-stress exposure. Operationalizing the concept of resilience in higher organisms, like mammals, has been difficult because it includes a range of many different phenotypic traits [26]. Our approach represents a novel way in understanding resilience in a living system, and we show how the inherent complexity of stress recovery can be exploited to predict the chance of survival. We anticipate that our finding will accelerate progress in the study of resilience of complex living systems, opening up new avenues of research in stress, aging, and disease across other species.

## Methods

### Strains and maintenance

The wild-type *C. elegans* strains N2 (Bristol) and CB4856 (Hawaii) were used in this study, as well as 54 CB4856 x N2 recombinant inbred lines (RILs; each line is a genetic mosaic with contributions of the two parental strains [9, 15, 16], and 47 CB4856 x N2 introgression lines (ILs; one individual locus of the CB4856 genome introgressed into an otherwise N2 genetic background [24]). Strains were maintained under standard culturing conditions on (9 cm diameter) Petri dishes with Nematode Growth Medium (NGM) containing *Escherichia coli* OP50 as food source [27]. To prepare populations for the start of an experiment, maintenance populations were chunked to fresh 9-cm NGM plates with food and kept at 20 °C for exactly 1 week to induce starvation. This was done to assure that all populations received the same treatment before the experiment.

### Lifespan under heat stress and control conditions

Starved populations were transferred to fresh NGM dishes seeded with *E. coli* OP50 by chunking and grown at 16 °C or 20 °C (depending on the desired growth rate) for 3–4 days to obtain proliferating populations. The populations were age synchronized by hypochlorite treatment according to standard protocols [27] and grown at 20 °C until the fourth larval stage was reached. At 47 h post age synchronization, the larvae were collected from the plates with M9 buffer, and 30–40 individuals were transferred to new NGM dishes containing 5-fluorodeoxyuridine [28]. At 48 h post age synchronization, the heat-stress-treated group was exposed to 35 °C for 4 h. Control and post-heat-stress conditions were set to 20 °C. Survival was scored every day by checking the response to touch with a picking needle. For each genotype, an average number of 31 animals were scored for each treatment.

For each genotype and treatment, the individual survival curves are reported in Additional file 4 with corresponding logrank statistics in Additional file 1: Table S6. For each genotype and treatment, we computed the average lifespan. The average effect of heat stress on lifespan (i.e., thermotolerance, reported in Fig. 4c) is the ratio between the average lifespan after heat stress and the average lifespan in the control.

### Transcriptome profile of heat stress, recovery, and developmental state

#### Data retrieval

Subsets of the transcriptomic profiles (measured with Agilent *C. elegans* (V2) Gene Expression Microarray 4X44K slides) used in this study have previously been described. These subsets include the developmental time series previously described in Snoek et al. [12], retrieved from Array Express under accession number E-MTAB-7019 [29], which includes 22 transcription profiles of N2 populations sampled in hourly intervals between 44 and 58 h post synchronization. The heat-stress time series was described in Jovic et al. [8], which includes 29 transcription profiles established after an exposure to 35 °C for 0, 0.5, 1, 2, 3, 4, 6, 8, or 12 h starting at 46 h after age synchronization (retrieved from ArrayExpress under accession number E-MTAB-5753 [30]). Transcription profiles of the RILs and ILs including the parental lines (N2 and CB4856) in control conditions (sampled 48 h post age synchronization), after a 2-h heat stress starting at 46 h post age synchronization, and after a subsequent 2-h recovery period were first presented by Snoek et al. ([9]; data retrieved from ArrayExpress, accession number E-MTAB-5779 [31]) and Sterken et al., ([25]; ArrayExpress, accession number E-MTAB-7424 [32]), respectively. A detailed overview of data sets and the according publications can be found in Additional file 1: Table S1.

#### Stress treatment and sampling for transcriptome analysis

The above-described transcriptome dataset was extended with a heat-stress time series for CB4856, and a recovery time series for N2 using protocols adapted from Snoek et al. [9, 12] and Jovic et al. [8]. Starved populations were transferred onto fresh NGM dishes seeded with *E. coli* OP50 by chunking and grown at 16 °C or 20 °C for 3–4 days depending on the desired growth rate to obtain gravid hermaphrodites. Age-synchronized populations were obtained by hypochlorite treatment according to standard protocols [27] and maintained at 20 °C until the begin of the heat-stress exposure of 35 °C starting at 46 h post age synchronization. For the CB4856 heat-stress time series, samples were taken after 0, 0.5, 1, 2, 4, and 6 h at 35 °C by rinsing the populations of the NGM plates with M9 buffer. For the N2 recovery time series, samples were transferred to 20 °C after 2, 3, 4, or 6 h at 35 °C. After 2 h of heat stress, samples were taken 0, 0.5, 1, 1.5, 2, 4, and 6 h into the recovery period. For 3, 4, or 6 h of heat stress, samples were taken in an hourly interval up to 4 h post-exposure. All samples were immediately flash frozen in liquid nitrogen at the time of collection and stored at − 80 °C until further use.

#### RNA isolation

mRNA was isolated from frozen samples using the Maxwell® 16 AS2000 instrument with a Maxwell® 16 LEV simplyRNA Tissue Kit (both Promega Corporation, Madison, WI, USA). The mRNA was isolated according to protocol with a modified lysis step as described in Snoek et al. and Jovic et al. Two hundred microliters homogenization buffer, 200 μl lysis buffer, and 10 μl of a 20 mg/ml stock solution of proteinase K were added to each sample. The samples were then incubated for 10 min at 65 °C and 1000 rpm in a Thermomixer (Eppendorf, Hamburg, Germany) before cooling on ice for 1 min. At this point, the samples were pipetted into the cartridges resuming with the standard protocol.

#### Sample preparation and scanning

For cDNA synthesis, labelling, and the hybridization reaction, the ‘Two-Color Microarray-Based Gene Expression Analysis; Low Input Quick Amp Labeling’ - protocol, version 6.0 from Agilent (Agilent Technologies, Santa Clara, CA, USA) was followed, starting at step 5. The Agilent *C. elegans* (V2) Gene Expression Microarray 4X44K slides were used in combination with an Agilent High-Resolution C Scanner using the recommended settings. Data was extracted with the Agilent Feature Extraction Software (version 10.7.1.1) following the manufacturers’ guidelines.

#### Data normalization and preparation

Microarray data were normalized using a within array normalization using a standard function of the R package limma (using “loess” method) [33]. The datasets were prepared for further analysis by removing genes with low average expression levels because of the level of noise they introduce into the data sets. Based on the bimodal distribution of gene expression levels (log2 intensity), a threshold was set at 4.5.

#### Derivation of axes

To derive quantitative axes that measure the transcriptional response to heat stress, four datasets were used: developmental time series, L4-stage RILs, heat-stress time series, and RILs in heat-stress at L4 stage. Each dataset was subsetted; one part was used to derive an axes and the other part was used to test the axes (see Additional file 1: Table S1). A principal component analysis (using R function prcomp()) was performed on the four separate, centered, and scaled data sets. In each case, the second principal axis captured the variation of interest resulting in the axis *D* (variation in development in time), axis *GD* (variation across genotypes in control conditions at 48 h), and two more axes capturing the variation within each of the two heat-stress datasets. This variation in gene expression levels within the heat-stress data sets is also caused by confounding influences such as development. *D* and *GD* were used to correct for the confounding influences and to isolate the stress-induced variation in time and between RILs, respectively. This was done by finding the axis H (GH), orthogonal to D (GD), that best explained the time variation of expression during heat stress. More specifically, we defined H = Ĥ − **(**Ĥ ·D**)** D, where Ĥ is the principal component axis obtained from the heat-shock data and **(**Ĥ ·D**)** is the scalar product between Ĥ and D. The rationale behind this choice is that the change in expression during heat stress could be affected by both stress response and development. This method provides a way to disentangle the two effects. Note however that the projection of the heat-stress (and recovery) data on the developmental axis do not show a time dependence and show small variation (see Additional file 2: Figure S3), strongly suggesting that the developmental changes during stress are small. A detailed description and explanation of the methods can be found in the supplementary methods (Additional file 2). The code needed to replicate all the results presented here can be found at https://github.com/jacopogrilli/resiliencevitality.git.

#### Using derived principal axes to measure stress response

By projecting the gene expression levels on the inferred axis, we can deduce where a particular sample/time-point places in comparison with the others. The projections on these axes display a clear time dependence, strongly suggesting that the axis capture the time variability of gene expression during time. Note, however, that this dependence does not correspond to any trivial properties about the actual dynamics of gene expression level. More explicitly, a monotonicity of the relation between the projection and time does not imply a monotonic dependence of gene expression levels on time [34].

#### Comparison with eQTL experiments

We compared the axes *H*, *D*, *GD*, and *GH* with expression QTL experiments in *C. elegans*. This analysis was conducted on re-mapped experiments downloaded from WormQTL [35, 36] and WormQTL2 [37]. These datasets consist of six different experiments representing 11 life-stage and temperature conditions: (i) L3-stage animals at 16 °C [15], (ii) L4-stage animals at 20 °C [9], (iii) 60-h-old young adults at 20 °C [21], (iv) 72-h-old adults at 20 °C [22], (v) L3-stage animals at 24 °C [15], (vi) late L3-stage animals at 24 °C [19], (vii) L4-stage animals grown at 24 °C [20], (viii) 96-h-old adults at 24 °C [20], (ix) 214-h-old adults at 24 °C [20], (x) L4-stage animals exposed to a 2-h 35 °C heat-shock [9], and (xi) L4-stage animals exposed to a 2-h 35 °C heat shock that have recovered for 2 h [9].

For all these experiments, mapped eQTL were obtained from WormQTL2 (based on WS258). We tested enrichment of the top 5% contributing genes of each of the four axes in genes with an eQTL in these experiments. We calculated the significance of the overlap using a hypergeometric tests. The *p* values were called significant at a Bonferroni-corrected threshold of *p* < 0.001 and an overlap of more than 10 genes.

#### Enrichment analysis

Enrichment analyses on the four axes (*H*, *D*, *GD*, and *GH*) were done using a hypergeometric test on the contributing genes. Enriched categories were filtered according to the following criteria: Bonferroni-corrected *p* < 0.05; size of the category, *n* > 3; and size of the overlap, *n* > 2.

We used the following databases: the WormBase [38] WS258 gene class names, anatomy terms, phenotypes, RNAi phenotypes, developmental stage expression, and disease-related genes [39]; the ModERN resource transcription-factor binding sites [40], which were mapped to transcription start sites (according to [41]). Additionally, we performed an enrichment analysis with DAVID 6.8 with the pre-defined selection of settings: Functional Categories (COG_Ontology, UP_keywords, Up_seq_feature), Gene_ontology (goterm_bp_direct, goterm_cc_direct, goterm_mf_direct), Pathways (KEGG_pathway), Protein_domains (Interpro, Pir_superfamiy, smart) [42, 43].

#### Analysis of polymorphisms between N2 and CB4856

Polymorphisms between N2 and CB4856 were obtained from Thompson et al. [16]. We tested enrichment of the top 5% contributing genes of each of the four axes in genes with polymorphisms related to different features (deletion of an exon, frameshifts, full deletion of a gene, located in the 5′ or 3′ regulatory region, affecting splicing, leading to gained or lost stops, in-frame insertions/deletions, and (non-)synonymous substitutions).

We calculated the significance of the overlap using a hypergeometric test. The *p* values were called significant at a Bonferroni-corrected threshold of *p* < 0.001 and an overlap of more than 10 genes.

## Supplementary information

**Additional file 1: **Table S1. Overview of microarray samples used in this study. Includes detailed information on the treatment conditions, previous publications, ArrayExpress accession numbers, and whether they were used for deriving the different axes (H, GH, D, GD). Table S2. Top 5% of genes contributing to the different axes. Table S3. Full output of the enrichment analysis using the webtool DAVID 6.8. Enrichment categories (using standard settings) include: Functional Categories (COG_Ontology, UP_keywords, Up_seq_feature), Gene_ontology (goterm_bp_direct, goterm_cc_direct, goterm_mf_direct), Pathways (KEGG_pathway), Protein_domains (Interpro, Pir_superfamiy, smart). Table S4. Additional enrichment output. The following resources were used: WormBase (https://wormbase.org/) version WS258 gene class names, anatomy terms, phenotypes, RNAi phenotypes, developmental stage expression, and disease-related genes, and the ModERN resource transcription-factor binding sites (http://epic.gs.washington.edu/modERN/). Table S5. Lifespan data. Columns X1-X51 gives the accumulative number of dead worms on day 1-51 post age-synchronization. Each row represents a different genotype. Control conditions are continuous 20°C; heat-stress animals were exposed to 35°C for 4h starting 46h after age-synchronization, after which they were returned to 20°C. Table S6. Lifespan statistics. Output of logrank test comparing lifespan in control conditions and after heat-stress. Tests were performed using the R package “survival” (vs. 2.42-6). The table also includes the FDR-adjusted *p*-values.

**Additional file 2:.** Background information on the methods used to derive the axes including supplementary figures S1-S9. Figure S1. Distribution of the logarithm of gene expression levels of developmental data. Figure S2. Analysis of principal components obtained from developmental data. Figure S3. Projections of N2 data on the second principal axis (developmental axis, D) vs. time. Figure S4. Components of the first principal axis inferred using a subset of the heat-stress vs. the corresponding average expression level. Figure S5. Projection on the second principal axis (heat-stress axis, H) vs. time. Figure S6. Projection of IL- and RIL-samples on the heat-stress axis H. Figure S7. Comparison between the variability of the projection on the heat-stress axis of RILs, ILs and N2. Figure S8. Projection of RILs and ILs data on the RIL axis. Fig S9 - Projection of RILs and ILs data on the RIL heat-stress axis, GH.

**Additional file 3: **Figure S10. Comparison with previous expression QTL studies. Heatmap comparing the top contributing genes of the axes *H, D, GD,* and *GH* with eQTL experiments from studies using *C. elegans* at various ages and treatments. Figure S11. Enrichment of the top contributors of axes *H, D, GD,* and *GH* with genes containing polymorphisms between N2 and CB4856. Figure S12. Projections of RIL data on GD and GH vs. the effect of a 4h heat-stress on lifespan. The effect of heat-stress on lifespan was given as the average lifespan in control conditions divided by average lifespan when exposed to a short heat-stress. Each point represents a different genotype. Correlations marked with a red cross were significant (i.e. Spearman; *p* < 0.05). Figure S13. Projections of IL data on axis GD and axis GH vs. the effect of heat-stress on lifespan. Figure S14. Projection of RIL data on D and H vs. the effect of heat-stress on lifespan. Figure S15. Projections of IL data on D and H vs. the effect of heat-stress on lifespan.

**Additional file 4.** PDF file containing 85 figures (i.e. one figure for each genotype) depicting survival curves in heat-stress and control conditions. Associated statistics output can be found in Additional file 1: Table S6.

## Data Availability

All data generated or analyzed during this study are included in this published article and its supplementary information files. All microarray datasets are available in the ArrayExpress database at EMBL-EBI ([44]) under the accession numbers E-MTAB-7007 [45], E-MTAB-7948 [46], E-MTAB-7019 [29], E-MTAB-5753 [30], E-MTAB-5779 [31], and E-MTAB-7424 [32]. Lifespan data is available in Additional file 1: Table S5.
