# Supplementary material for: Transcriptome resilience predicts thermotolerance in Caenorhabditis elegans
Source: BMC Biol. 2019 Dec 10;17:102. doi: 10.1186/s12915-019-0725-6 (PMC6905072; doi:10.1186/s12915-019-0725-6)
Supplement: Supplementary file 2 — Additional file 2:. Background information on the methods used to derive the axes including supplementary figures S1-S9. Figure S1. Distribution of the logarithm of gene expression levels of developmental data. Figure S2. Analysis of principal components obtained from developmental data. Figure S3. Projections of N2 data on the second principal axis (developmental axis, D) vs. time. Figure S4. Components of the first principal axis inferred using a subset of the heat-stress vs. the corresponding average expression level. Figure S5. Projection on the second principal axis (heat-stress axis, H) vs. time. Figure S6. Projection of IL- and RIL-samples on the heat-stress axis H. Figure S7. Comparison between the variability of the projection on the heat-stress axis of RILs, ILs and N2. Figure S8. Projection of RILs and ILs data on the RIL axis. Fig S9 - Projection of RILs and ILs data on the RIL heat-stress axis, GH. [file 12915_2019_725_MOESM2_ESM.pdf]

Supplementary Information

Contents

S1. Singular Value Decomposition and Principal Component Analysis 2

S2. Principal axis for N2 3

A. Derivation of the developmental axis D 3

B. Derivation of the heat-stress axis H 6

S3. RILs data 9

A. Projection of RILs data on H 9

B. Derivation of the genetic heat-stress axis, GH 10

S1. SINGULAR VALUE DECOMPOSITION AND PRINCIPAL COMPONENT ANALYSIS

In this section, we describe how we obtained the principal axis from the data. After the canonical corrections of the microarray data (see Methods), we can arrange our data in a matrix $\mathbf{X}$, with G rows and S columns, where G is the number of spots in the microarray ($G = 45220$) and S is the number of samples. The element X_{sg} represents the logarithm of the intensity ("expression level" henceforth) obtained from the micro-array data after the procedures described in the Methods.

Since the variability in both the average and the variance of intensities across the samples might not be due to biological differences, we rescale the data such that the average expression level is equal to 0, and the variance is equal to 1. The transformation is

$$\tilde{X}_{sg} = \frac{X_{sg} - (1/G)\sum_g X_{sg}}{\sqrt{(1/G)\sum_g X_{sg}^2 - \left((1/G)\sum_g X_{sg}\right)^2}} \ , \tag{1}$$

such that

$$\frac{1}{G}\sum_g \tilde{X}_{sg} = 0 \ , \tag{2}$$

and

$$\frac{1}{G}\sum_g \tilde{X}_{sg}^2 = 1 \ . \tag{3}$$

In full generality, the elements of the matrix $\tilde{\mathbf{X}}$ can be written as

$$\tilde{X}_{sg} = \sum_{\alpha=1}^{S} \lambda_\alpha w_s^\alpha v_g^\alpha \ , \tag{4}$$

where

$$\sum_{s=1}^{S} w_s^\alpha w_s^\beta = \sum_{g=1}^{G} v_g^\alpha v_g^\beta = \delta_{\alpha\beta} \ , \tag{5}$$

and $\delta_{\alpha\beta}$ is the Kroneker delta (equal to one if $\alpha = \beta$, and zero otherwise). The factorization of the matrix $\tilde{\mathbf{X}}$ shown in equation 4 is called the Singular Value Decomposition (SVD) of the matrix $\tilde{\mathbf{X}}$. The vectors $\mathbf{w}^\alpha$ and $\mathbf{v}^\alpha$ are the singular vector of the matrix $\tilde{\mathbf{X}}$—specifically, one can define the $S \times S$ matrix $\tilde{\mathbf{X}}^T\tilde{\mathbf{X}}$ which has eigenvalues λ_α^2 and eigenvectors $\mathbf{w}^\alpha$ (note that this matrix is positive semi-definite, as it can be seen as a covariance matrix). On the other hand, the $G \times G$ matrix $\tilde{\mathbf{X}}\tilde{\mathbf{X}}^T$ has S eigenvalues equal to λ_α^2 with eigenvectors $\mathbf{v}^\alpha$, while the other $G - S$ eigenvalues are all equal to zero.

The entries of the matrix $\tilde{\mathbf{X}}^T\tilde{\mathbf{X}}$ are defined as

$$(\tilde{\mathbf{X}}^T\tilde{\mathbf{X}})_{ss'} = \sum_g \tilde{X}_{sg}\tilde{X}_{s'g} \ . \tag{6}$$

Because of the relation in equation 3, the diagonal elements are all equal to one, while the off diagonal coefficients represent the correlations between the expression pattern of two samples. The diagonalization of this matrix and $\tilde{\mathbf{X}}\tilde{\mathbf{X}}^T$ allows to identify independent combinations of genes that explain most of the variation observed in the data.

Principal Component Analysis employs singular value decomposition (i.e., the factorization shown in equation 4) to identify the components that explain most of the variation in the data. Since all the values λ_α are positive, one can always ordered them to have $\lambda_1 > \lambda_2 > \cdots > \lambda_S \geq 0$. The first principal component (often called PC1) is then simply w_s^1, the second one w_s^2 and so on. The fraction of variance explained by the kth component can be simply expressed in terms of the eigenvalues

$$\frac{\lambda_k^2}{\sum_{\alpha=1}^{S} \lambda_\alpha^2} \ . \tag{7}$$

The vectors $\mathbf{v}^\alpha$ parallel the information given by the vectors $\mathbf{w}^\alpha$. Two samples , s_1 and s_2, have similar expression patterns if the entries s_1 and s_2 of the first principal components $\mathbf{w}^1$, $\mathbf{w}^2$, ... have similar values. In the same way, two genes g_1 and g_2 have similar expressions *across* samples, in the corresponding entries of $\mathbf{v}^1$, $\mathbf{v}^2$, ... are close. It is important to remember that, if the vector $\mathbf{v}^\alpha$ is known, the corresponding vector $\mathbf{w}^\alpha$ can be obtained from

$$\sum_g \tilde{X}_{sg} v_g^\alpha = \lambda_\alpha w_s^\alpha \ . \tag{8}$$

S2. PRINCIPAL AXIS FOR N2

In this section, we explain how we obtain the developmental axis, D, and the heat-stress response axis, H, for N2.

A. Derivation of the developmental axis D

The developmental data of the N2 strain were obtained in two batches, *set*11 (12 data points) and *set*13 (8 data points), see methods. We used only the data from the batch *set*11 to infer the principal axis $\mathbf{v}^\alpha$. Once the axis is known, one can obtain the corresponding component by projecting the data over these vectors (see equation 7). In this section, we show that—using only the gene expression levels of one batch (without using a-priori information on the developmental stage of individual samples)—we are able to describe the change in expression due to development of all the other samples in the full dataset.

As explained in section S1, we consider the data matrix $\tilde{\mathbf{X}}$, whose entry $\tilde{X}_{sg}$ represents the normalized logarithm of the expression level of gene g in sample s.

Figure S1 shows that the distribution of gene expression levels across different samples is bimodal in both batches. For all samples, the minimum of the distribution between the two maxima occurs at an expression level around 4.5. Since genes that have low expression are noisier, we removed, for the purposes of the derivation of the axis, all

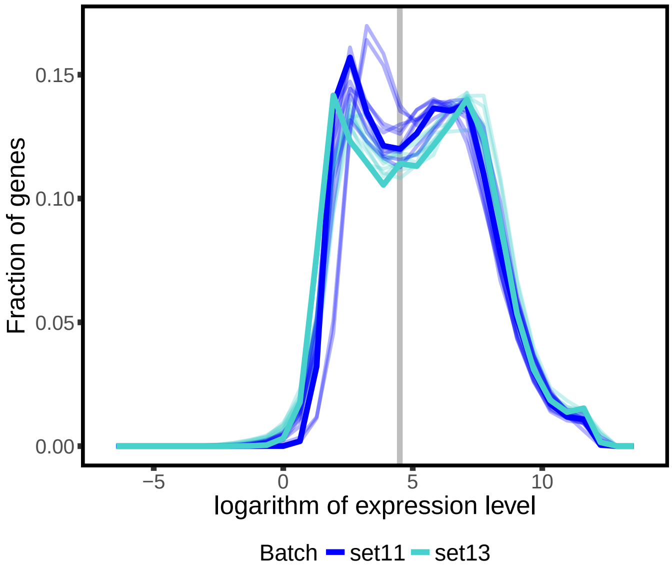

Supplementary Figure S1: Distribution of the logarithm of gene expression levels of developmental data. Each curve in the background corresponds to a sample, while the two solid curves mark the average over the samples of a given batch (dark blue for $set11$ and light blue for $set13$). While different samples correspond to different stages in development, the distribution is quite robust and is always bimodal. The gray line correspond to a value of the logarithm of the expression level equal to 4.5, which is the value we use as a threshold between highly- and lowly-expressed genes.

the genes whose average expression across the samples of batch $set13$ was lower than 4.5. In practice, we set the corresponding entries of v_g^α to zero.

Using the genes with average log-expression larger than 4.5, we removed mean and variance obtaining the data matrix $\tilde{\mathbf{X}}$ as described in section S1. Using only the samples from $set13$, we performed the singular value decomposition obtaining the principal axis $\mathbf{v}^\alpha$. In order to obtain the k-th principal component of any sample from any batch, we need to project the gene expression data on the axis $\mathbf{v}^k$. In other words, if $\tilde{X}_{sg}$ is the logarithm of the expression of gene g in sample s, the projection of sample s on the k-th axis is given by $\sum_g \tilde{X}_{sg} v_g^k$.

The first principal axis $\mathbf{v}^1$ is strictly related to the heterogeneity of expression levels among genes. Figure S2 shows that the entry of the principal vector is correlated with the average expression of genes. In particular, the relation is perfectly linear for batch $set13$, which is the one used to infer the axis. The first principal axis measures therefore the average expression and not the difference between samples and their change in time. Figure S2 shows that the second principal axis, on the other hand is not correlated with the average expression, implying that this vector captures other structural properties of the data.

Figure S3A shows that the projection of the data on the second principal axis has a clear trend with time (*i.e.* developmental age measured in hours after age-synchronization, see methods for details). While the previous Figure S2C shows that the third and fourth principal component are also influenced by time-dependent expression variation, the

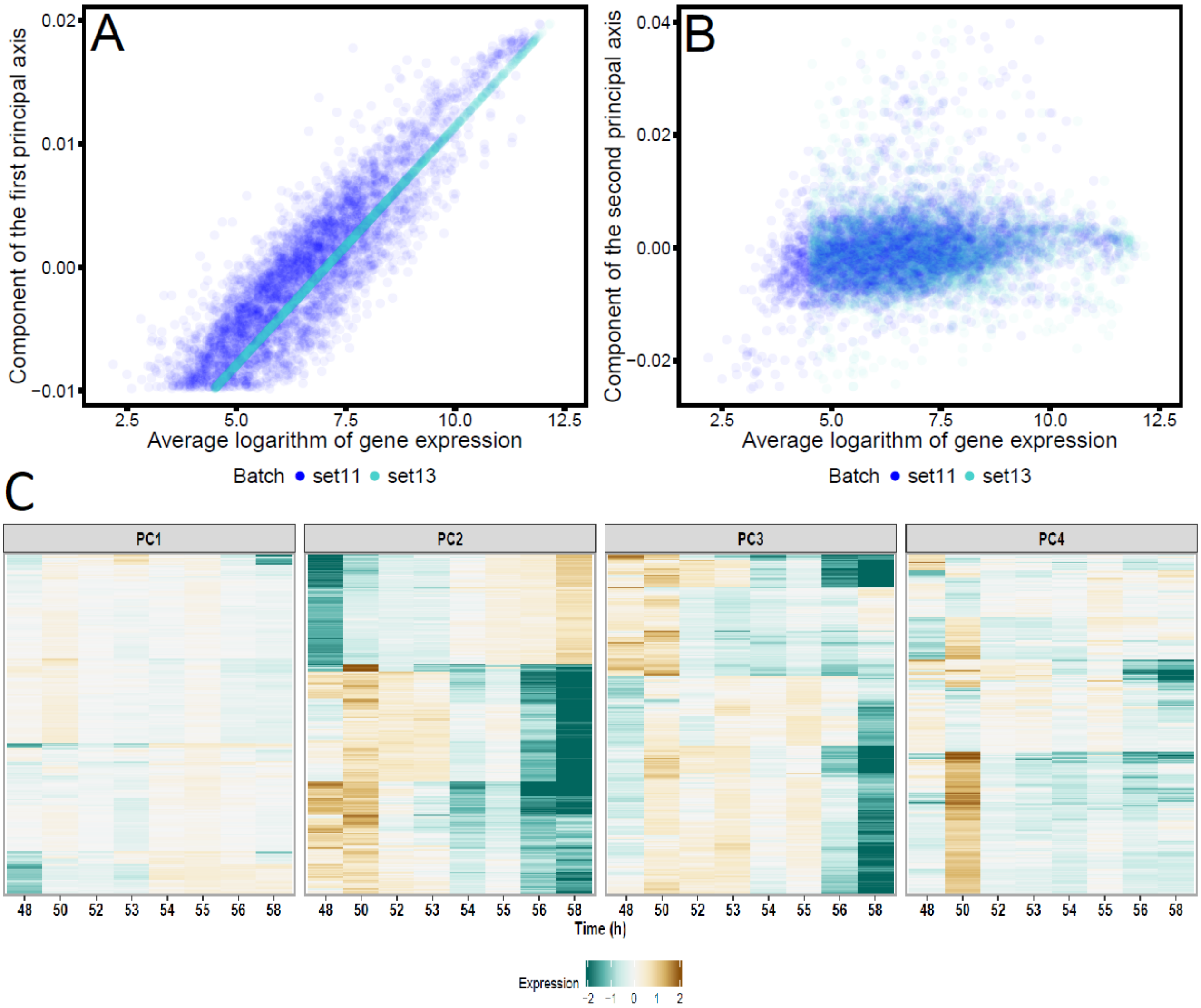

Supplementary Figure S2: Components of the first (panel A) and second (panel B) principal axis inferred using a subset of the developmental transcriptome data vs. the corresponding average expression level. The two axes were inferred using only the data from batch *set*13 sampled during unperturbed development. Panel A shows that the component of the first axis perfectly correlates with the average expression level of the corresponding gene for *set*13. This observation implies that the first principal axis only summarizes the average expression level of different genes and does not include any information on the dynamics of gene expression. Panel B shows that instead the second principal axis components are not correlated with average gene expression. In Panel C, gene expression dynamics during development in *set*13 of the top 5 percent of contributing genes for each component is shown in a heatmap. For each gene, expression levels were normalized to the average across all time points and log2 transformed. It can be seen that PC2 captures monotonic and non-monotonic time-dependent expression changes. Also genes contributing to PC3 and PC4 have time-dependent expression changes.

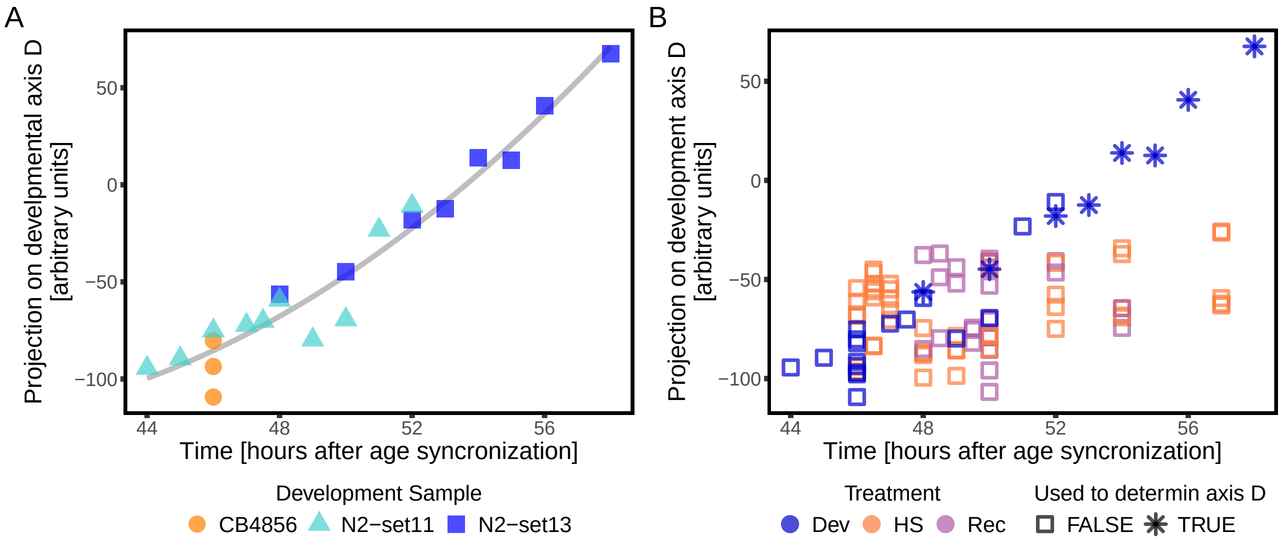

Supplementary Figure S3: Projection on the second principal axis (developmental axis, D) vs. time. Panel A: The projection on the developmental axis shows a clear dependence on time. It also shows that the axis, which was inferred using only the data of one genotype (N2) from one batch (*set*13), also describes the dynamics of another batch (N2, *set*11) and other genotype (CB4856), showing that the developmental axis also describes the expression dynamics of other strains. Panel B: Projection of unperturbed, perturbed and recovery data on the developmental axis D vs. time. Different colors correspond to different treatments (blue are unperturbed worms, orange corresponds to the heat-stress data and purple is recovery data). Only the data points marked with a star were used to determine the two axes, while the square symbols correspond to data that was not used in the inference process.

results shown in Figure S3A strongly suggest that the complex combination of monotonic and non-monotonic gene expression dynamics during development are efficiently reduced and captured by the second principal component and directly relates to the transcriptional developmental stage of the samples used in the analysis. It should be noted that the developmental axis, D, is also relating to the developmental timing of other samples within other batches, and also for other genotypes (*e.g.* CB4856, see Figure S3A), even though it was obtained using only the samples of batch *set*13. In addition to the developmental data, Figure S3B) shows the projection of N2-data collected during two further treatments (heat-stress, and recovery; see the following sections, and Methods for details) on the developmental axis D vs. time. Recovery samples do not return to unperturbed stress patterns, indicating that animals have not fully recovered from the 2-hour heat-stress within the observed recovery period.

B. Derivation of the heat-stress axis H

To produce the time-series profiling the expression levels during the response to heat-stress, N2 populations were exposed to 35°C for different durations (see methods for more details). Data were collected in 7 batches. In order to

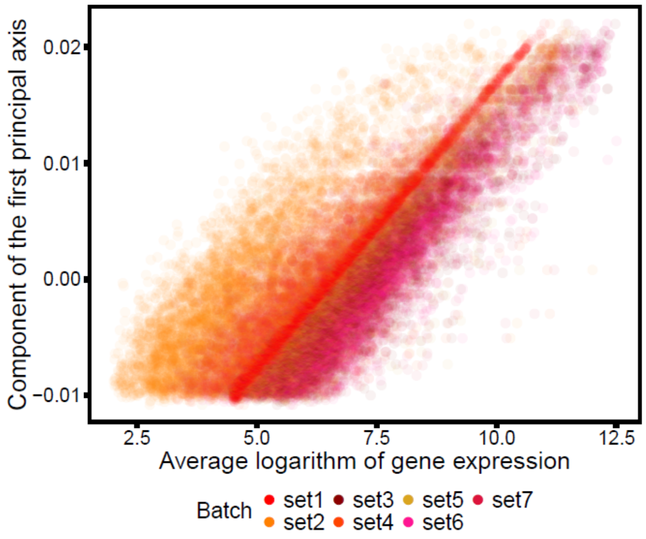

Supplementary Figure S4: Components of the first (panel A) principal axis inferred using a subset of the heat-stress vs. the corresponding average expression level. The axis was inferred using only the heat-stress data from batch $set1$. The component of the first axis correlates perfectly with the average expression level of the corresponding gene for $set1$, and it correlates strongly with average expression for the other batches. As such, the first principal axis only summarizes the average expression of different genes and does not include any information on the dynamics of gene expression.

obtain the heat-stress axis, we applied the same procedure as described in section S2 A.

First, we normalized the data, obtaining the matrix $\tilde{\mathbf{X}}$. Then, we derived the principal axis only considering the batch $set1$ (10 data points out of a total 39 heat-stressed N2 samples) and only the genes which had a log expression level averaged over the samples within batch $set1$ higher than 4.5.

The components of the first principal axis determined with this procedure are again correlated with the average gene expression and do not capture the information on the effect of different heat-stress perturbations (see Figure S4). As in the case of the developmental axis, we expect the second principal axis to be related to the time elapsed since the beginning of the perturbation. While the worms were exposed to heat-stress starting at a given age (*i.e.*, 46h post age-synchronization), development partially continues during heat-stress [Snoek *et al.*, 2017]. Therefore different exposure durations to heat-stress also correspond to different developmental ages. In other words, the observed effect on worms exposed to a heat-stress for different times is the combined effect of heat-stress response and development. In order to disentangle the effect of development from the effect of heat-stress, we considered the second principal axis found from $set1$ and we removed from it its projection on the developmental axis. The heat-stress axis, H, found in this way is orthogonal (and therefore independent) to the developmental axis, and captures only the effect of different heat-stress exposures.

Figure S5A shows the projection on the heat-stress axis of individual populations exposed for different lengths of

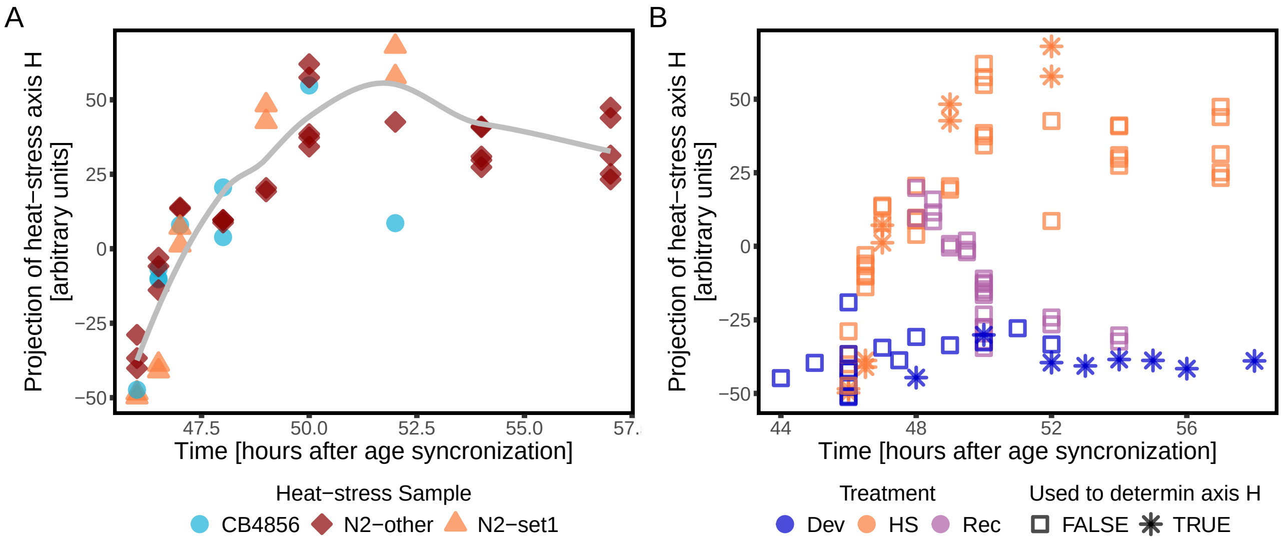

Supplementary Figure S5: Projection on the second principal axis (heat-stress axis, H) vs. time (note that heat-stress was started at 46 hours). Panel A: The projection on the heat-stress axis shows a clear dependence on time, suggesting that the complex multi-dimensional dynamics of gene expression during heat-stress can be efficiently reduced to a single variable. The axis was inferred using only the data from one batch ("*set*1"), but it also describes the dynamics of other batches (6 other separate batches shown as "N2-other" in the figure) as well as another strain (CB4856), showing that the heat-stress axis H also recapitulates the heat-stress response of other strains. Panel B: Projection of unperturbed, perturbed and recovery data on the heat-stress axis H vs. time. Different colors correspond to different treatments (blue are unperturbed worms, orange corresponds to the heat-stress data and purple is recovery data). Only the data points marked with a star were used to determine the axes, while the square symbols correspond to data that was not used in the inference process.

time—demonstrating how the axis is related to the heat-stress exposure duration. It should be noted that the axis was obtained using only a small sub-set of data points in *set*1, but it well describes the heat-stress response of all data points within the other sets. Moreover, the axis also describes the effect of heat-stress on the gene expression of other strains than N2 (*e.g.* CB4856; Figure S5A). Therefore, the heat-stress axis H provides a robust and quantitative measure of the heat-stress response across different genotypes. Figure S5B shows the projection of N2-data collected during two further treatments (development, and recovery) on the heat-stress axis, H, vs. time. The recovery data was obtained from N2 populations that were exposed to a 2-hour heat-stress (at 35°C degrees), after which they were put back to 20°C degrees. The projection of the recovery data on the heat-stress axis shows that H also describes the recovery dynamics after perturbation. This is notable, given that we did not use the recovery data to infer the heat-stress axis, H, nor to infer the developmental axis, D.

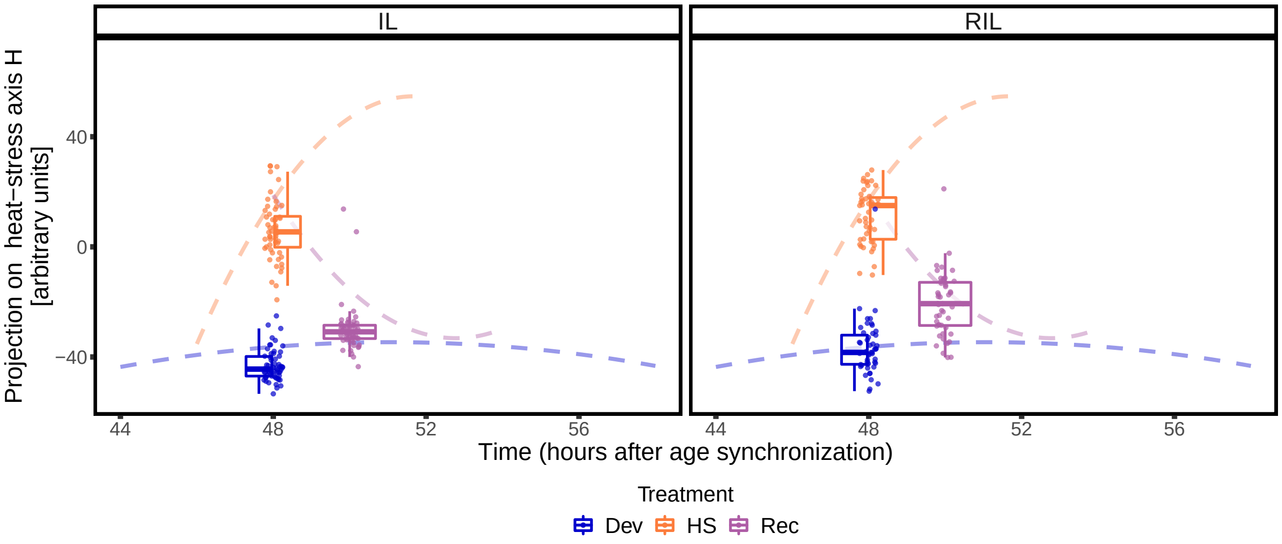

Supplementary Figure S6: Projection of IL- and RIL-samples on the heat-stress axis H. Different colors correspond to different treatments (blue is for unperturbed worms at 48h post-age synchronization, orange for heat-stressed samples after 2h at 35°C, and purple for recovering worms measured 2h after the heat-stress). Each point represents a genotype measured within a treatment-category; boxplots were added to illustrate the distributions in these measurements. The dashed lines in the background present the average N2 projections as a reference.

S3. RILS DATA

A. Projection of RILs data on H

To take a deeper look at genetic variation in the heat-stress response, we used gene expression profiles of a genetically diverse set of recombinant inbred lines (RILs) and introgression lines (ILs) in unperturbed development, after 2 hours heat-stress, and after 2 hours of recovery (see methods for more details). Figure S6 shows the projection of the RILs and ILs data on the heat-stress axis. RILs and the ILs samples have similar projections on H before, during and after the heat-stress. Moreover, the values of their projections is not particularly different from the values of the parental lines N2 and CB4856.

In order to explore the possible sources of the variability between RILs and ILs in their projections, we compared it with the variability observed for N2 across replicates of the same experiment. For each experiment, we computed the standard deviation of the projection on the heat-stress axis of N2 with the one obtained by sampling at random an equal number of RILs or ILs. Figure S7 shows that the variability of the ILs and RILs is higher, lower or comparable than the one of N2, depending on the treatment. This suggests that there is not a detectable genetic signal on the projection on the heat-stress axis, and that the variability of the projection is most likely due to variability in the conditions and noise rather than biological differences.

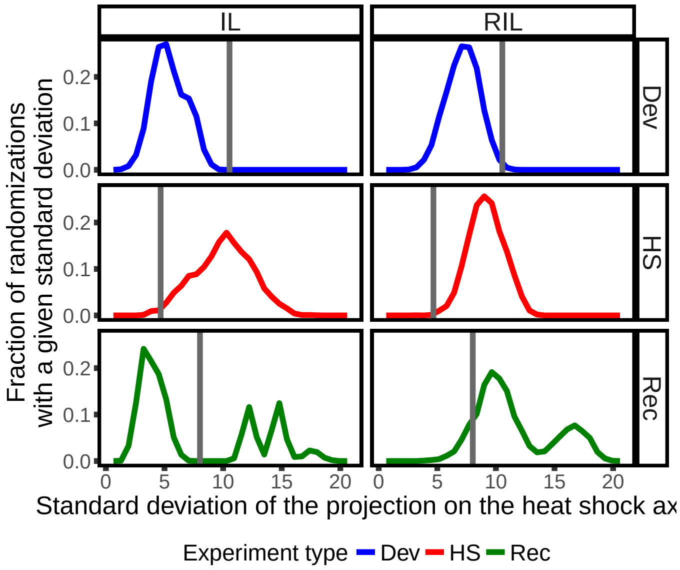

Supplementary Figure S7: Comparison between the variability of the projection on the heat-stress axis of RILs, ILs and N2. For each condition, we considered the replicates obtained with the N2 strain and measure the standard deviation of their projection across replicates (vertical gray bar). These values quantify the variability in the projection under different conditions. For each treatment, we then draw at random a number of RILs (or ILs) equivalent to the number of replicates available for N2 and measure the standard deviation of the random sample. By repeating the sampling 5000 times, we obtain a distribution of standard deviations (colored lines). The overlap between these distributions (colored lines) and the variability across replicates of the N2 strain quantifies whether genetic differences are responsible for the variability between RILs and ILs. The variability of RILs and ILs is comparable, resulting slightly larger in the RILs for the recovery experiment. In the case of unperturbed worms, the variability within N2 is larger than the variability between RILs. The projection obtained during heat-stress is instead more variable for RILs and ILs. The distribution of standard deviation for the random sampling of the recovery experiment is bimodal, due to the presence of outliers in the RILs and ILs, and their variability is comparable with the variability of N2.

B. Derivation of the genetic heat-stress axis, GH

As shown in section S3 A the variability of the projection on the heat-stress axis between RILs and ILs is not particularly different from the variability observed across replicates of the N2 strains (see Figure S7). This is not surprising, given the method used to infer the heat-stress axis. This axis was in fact inferred using only the N2 strains under different exposure to heat-stress. It is therefore reasonable to think that both the variability between genotypes at the same time point is due to fluctuations of the conditions or other sources of noise and not due to biological differences between organisms.

In order to explore the intrinsic differences between different genotypes, we applied a similar method to the one explained in section S2 B. Using only the RILs data taken after 48h of unperturbed development (47 data points) we performed a PCA and isolated the second principal axis. This axis (RILs axis in the following) is the one that

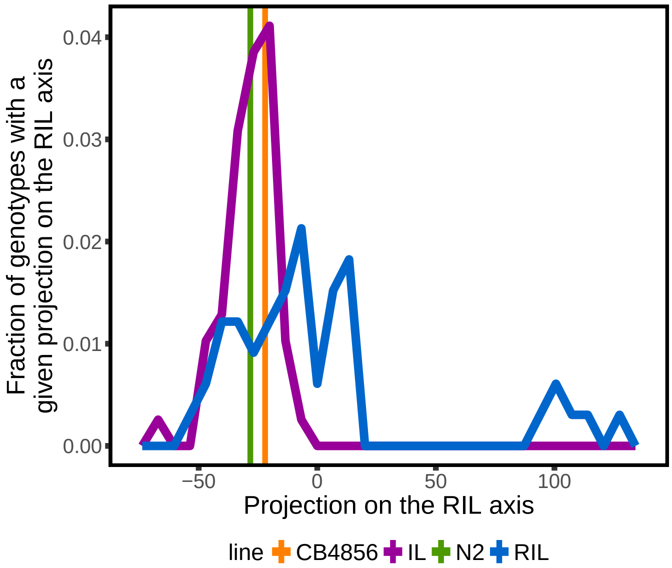

Supplementary Figure S8: Projection of RILs and ILs data on the RIL axis. The two vertical bars represent the projection of CB4856 (orange) and N2 (purple), averaged over replicates. The two lines are the distribution of the projections of RILs (blue line) and ILs (green line) on the RILs axis. The RILs axis was obtained as the second principal axis from the RILs data at 48h. All the worms considered in this figures were collected after growing under control conditions for 48h at 20°C degrees.

explains most of the variation between RILs at 48h in control conditions (20°C). Figure S8 shows the distribution of projections on this axis of RILs (used to infer the axis), but also of ILs and the two parental lines (N2 and CB4856). As expected, ILs show lower variability in the projection on this axis.

In order to measure the difference in the response to perturbations between different RILs, we then used the data obtained after a two hours heat-stress to obtain the principal axes. The second principal axis is the one containing the information on the different responses to heat-stress. It is also related to other differences between RILs and not exclusively to their response to heat-stress. These other differences are the ones contributing to the RIL axis obtained using the expression before heat-stress. We obtained therefore a RILs heat-stress axis by removing the RILs axis from the second principal axis obtained with the heat-stress data (see section S2 B).

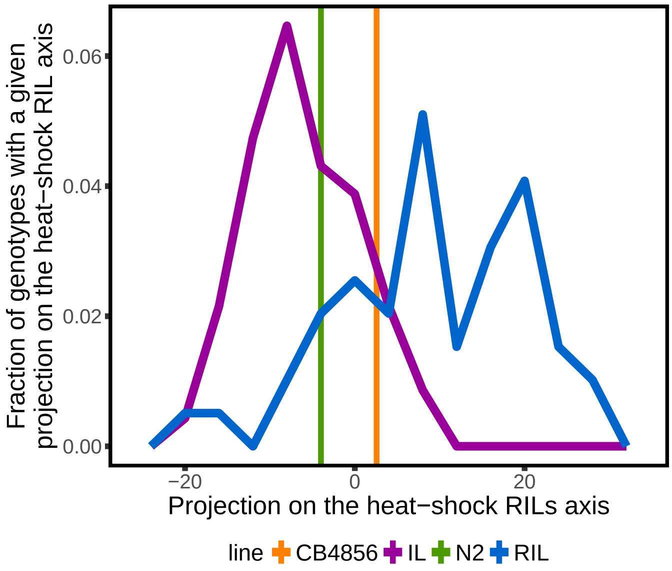

Supplementary Figure S9: Projection of RILs and ILs data on the RIL heat-stress axis, GH. The two vertical bars represent the projection of CB4856 (orange) and N2 (purple), averaged over replicates. The two lines are the distribution of the projections of RILs (blue line) and ILs (green line) on the RILs axis. The RILs axis was obtained as the second principal axis from the RILs data at 48h. All the worms considered in this figures were collected after 48h and grown at 20 degrees.
