# Supplementary figures and images for: Transcriptome resilience predicts thermotolerance in Caenorhabditis elegans
Source: BMC Biol. 2019 Dec 10;17:102. doi: 10.1186/s12915-019-0725-6 (PMC6905072; doi:10.1186/s12915-019-0725-6)

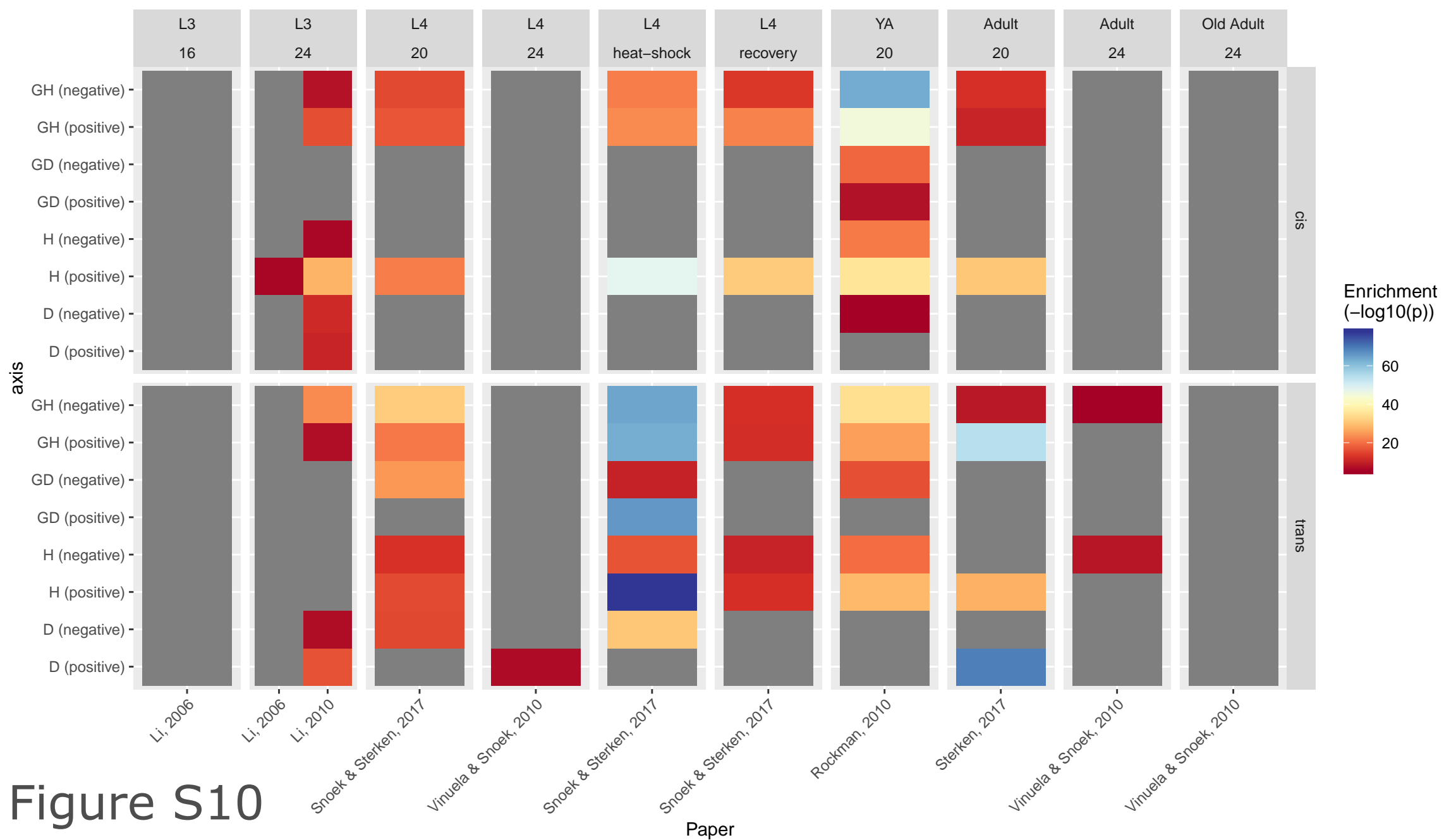

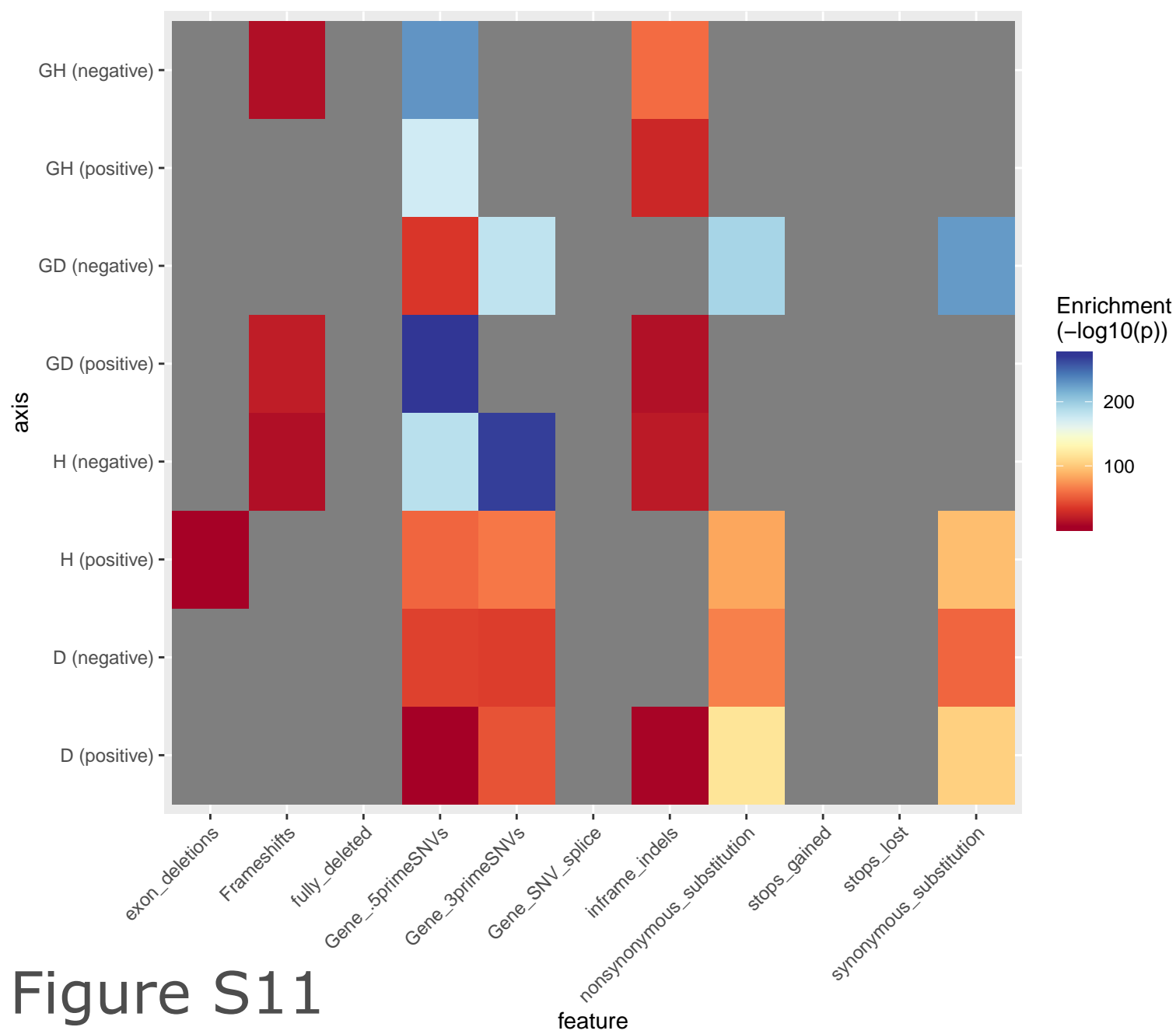

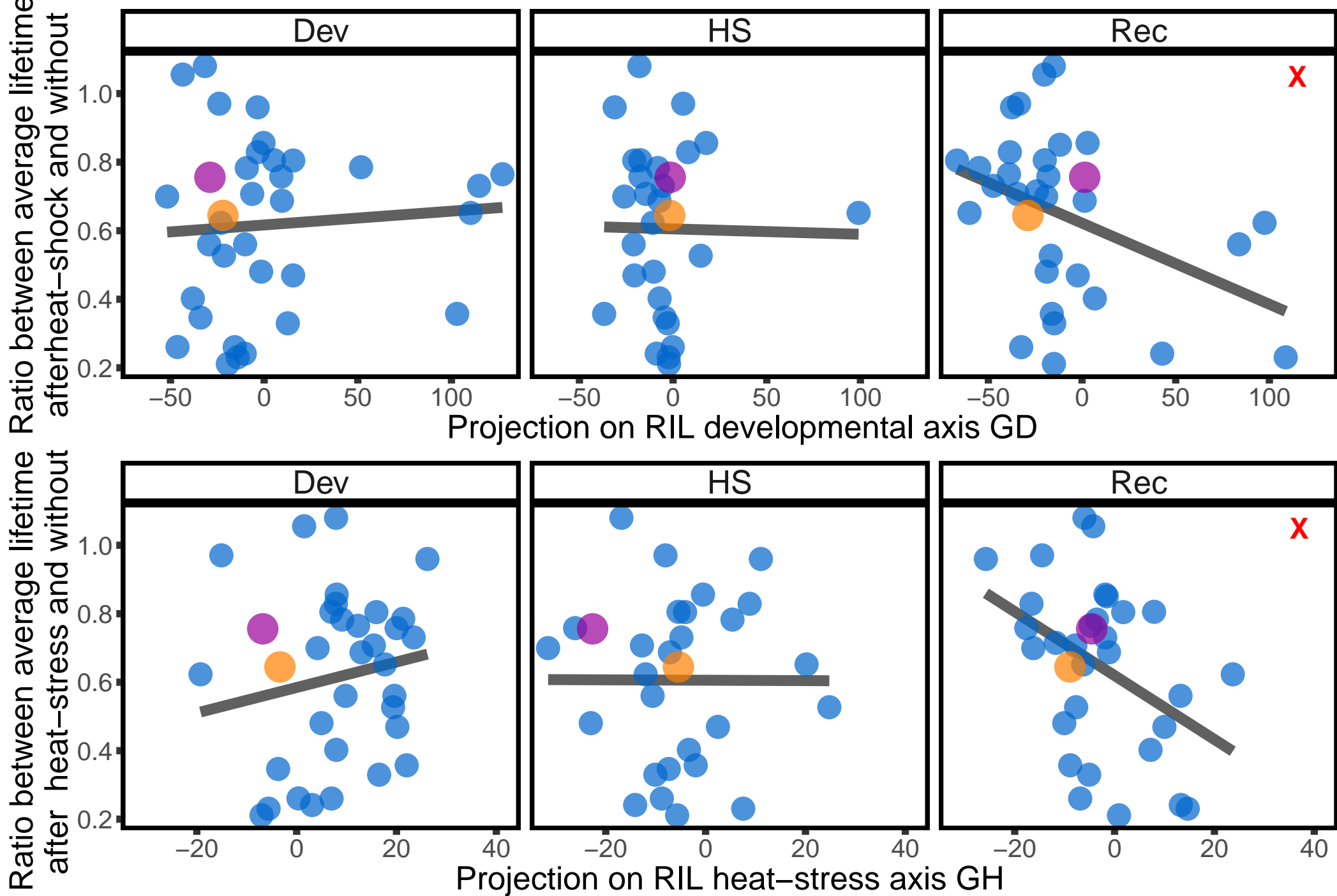

Figure S12

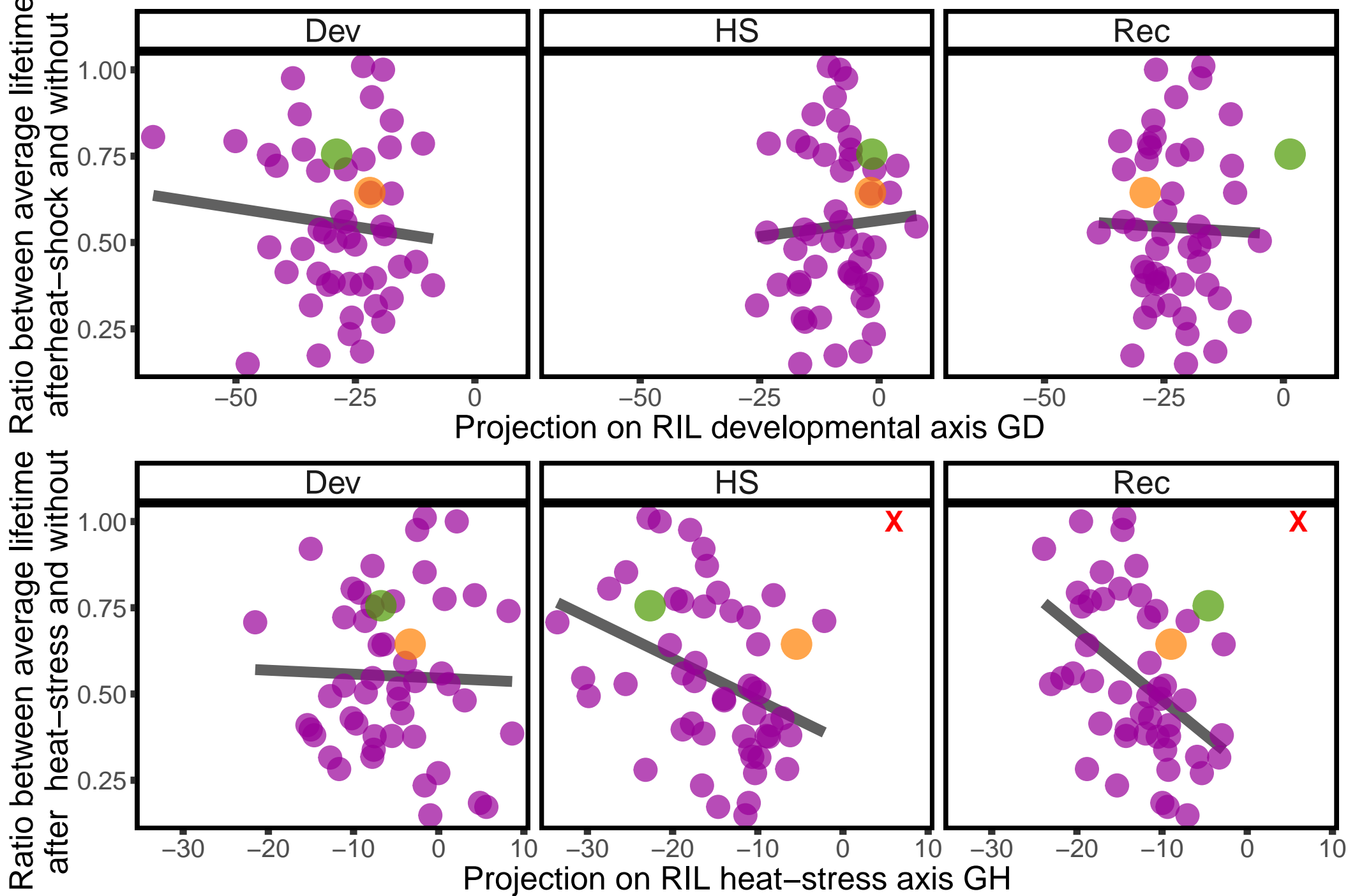

Figure S13

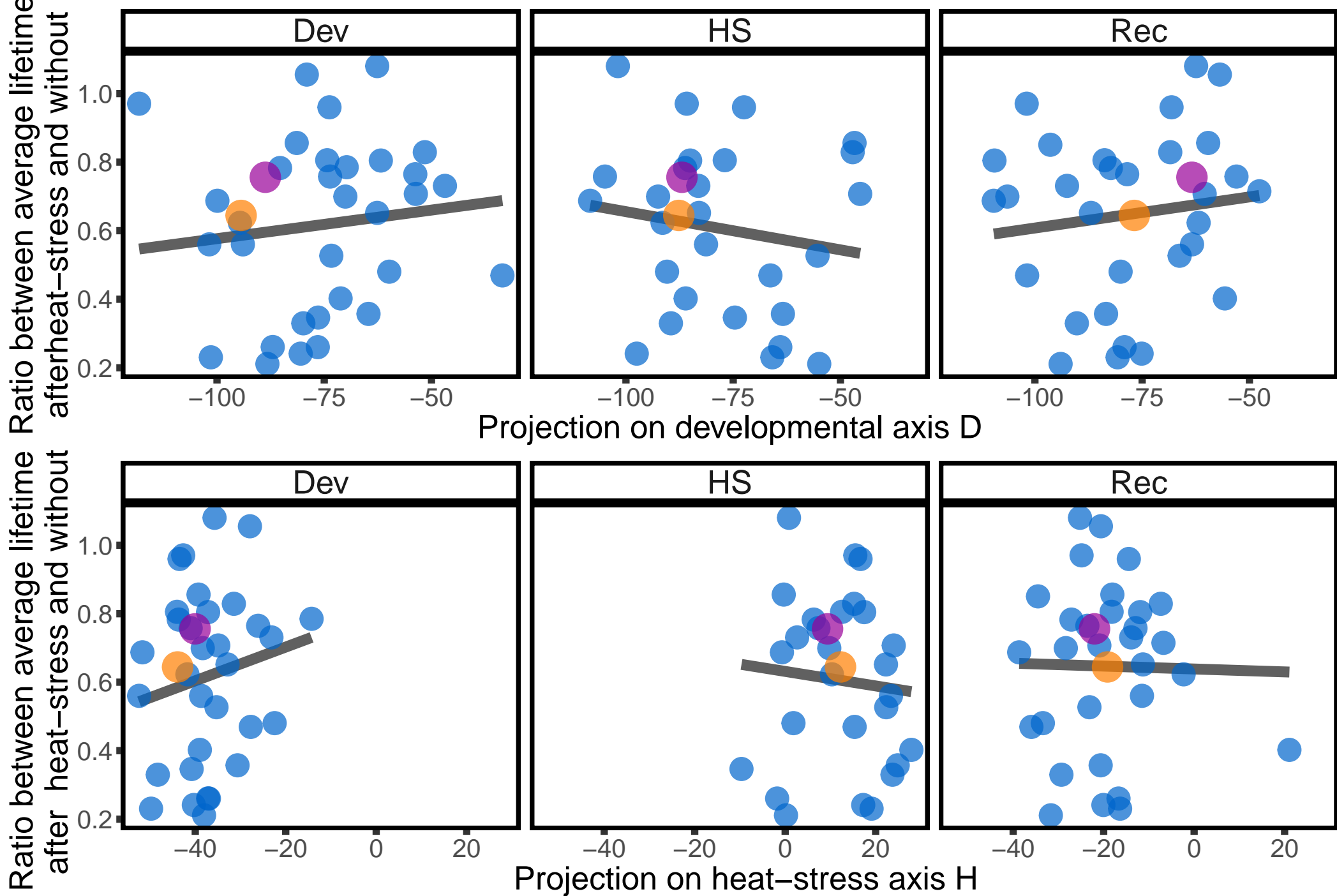

Figure S14

line ● CB4856 ● N2 ● RIL

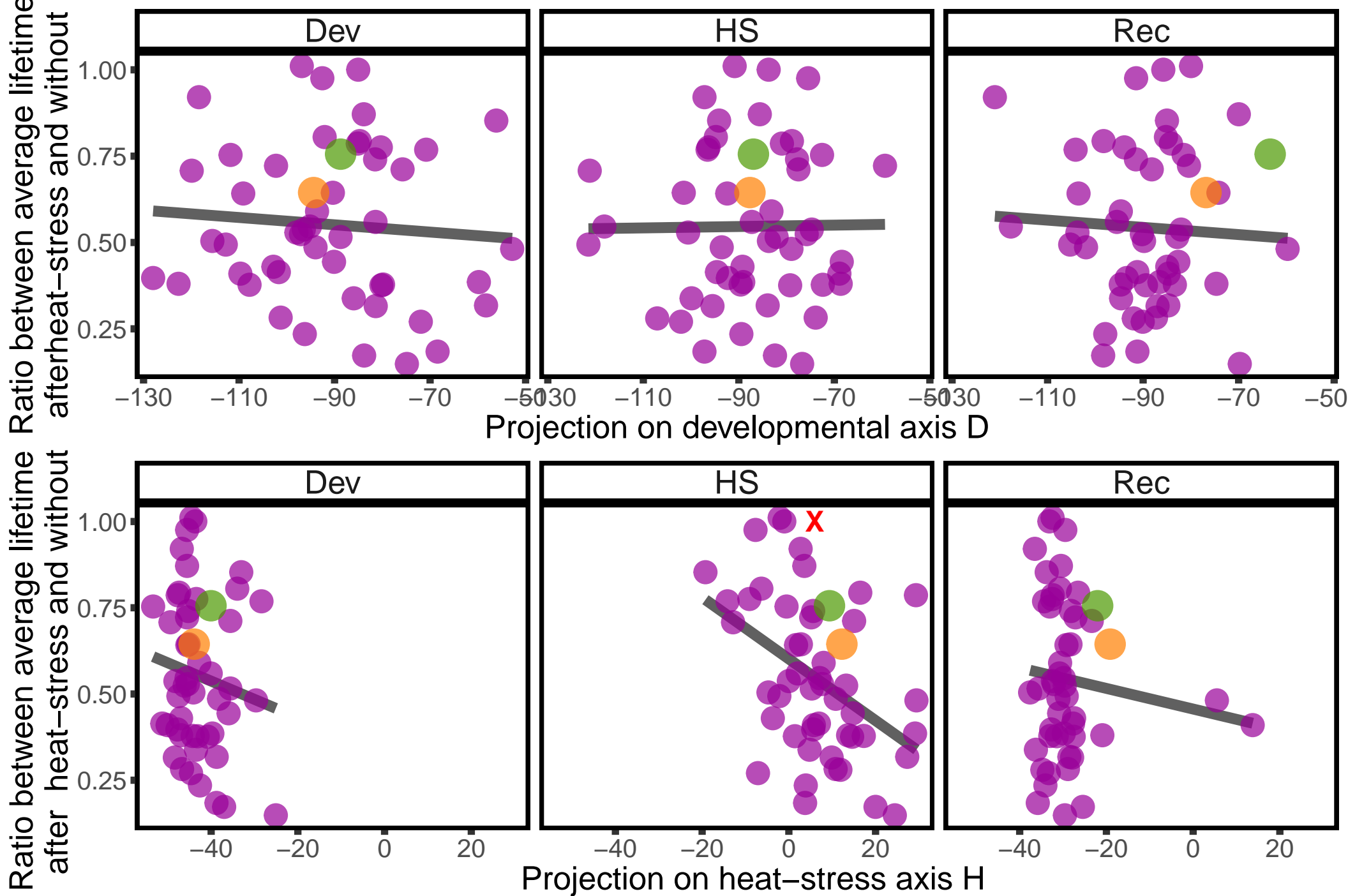

Supplement: Supplementary file 3 — Additional file 3: Figure S10. Comparison with previous expression QTL studies. Heatmap comparing the top contributing genes of the axes H, D, GD, and GH with eQTL experiments from studies using C. elegans at various ages and treatments. Figure S11. Enrichment of the top contributors of axes H, D, GD, and GH with genes containing polymorphisms between N2 and CB4856. Figure S12. Projections of RIL data on GD and GH vs. the effect of a 4h heat-stress on lifespan. The effect of heat-stress on lifespan was given as the average lifespan in control conditions divided by average lifespan when exposed to a short heat-stress. Each point represents a different genotype. Correlations marked with a red cross were significant (i.e. Spearman; p < 0.05). Figure S13. Projections of IL data on axis GD and axis GH vs. the effect of heat-stress on lifespan. Figure S14. Projection of RIL data on D and H vs. the effect of heat-stress on lifespan. Figure S15. Projections of IL data on D and H vs. the effect of heat-stress on lifespan. [file 12915_2019_725_MOESM3_ESM.pdf]

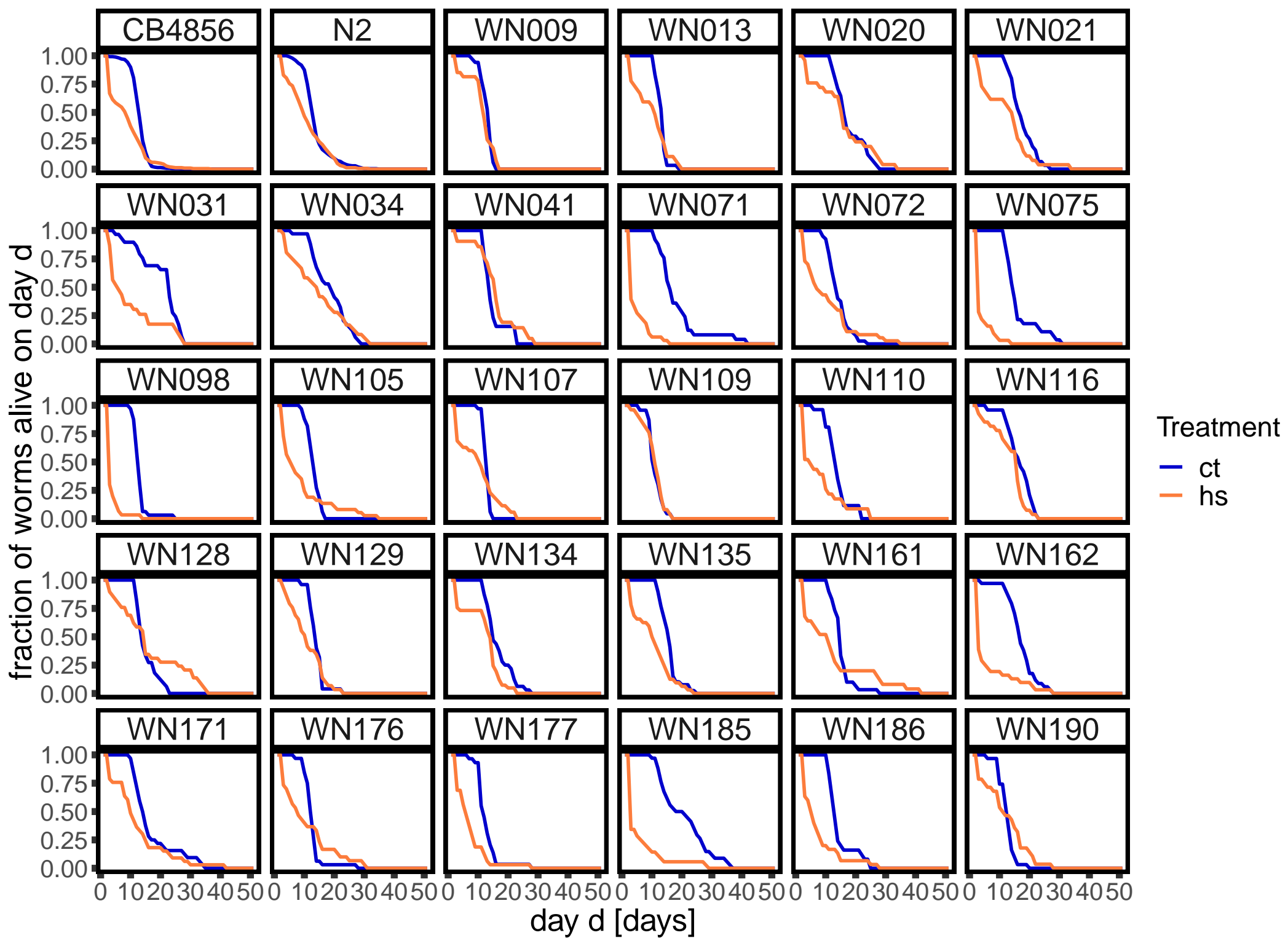

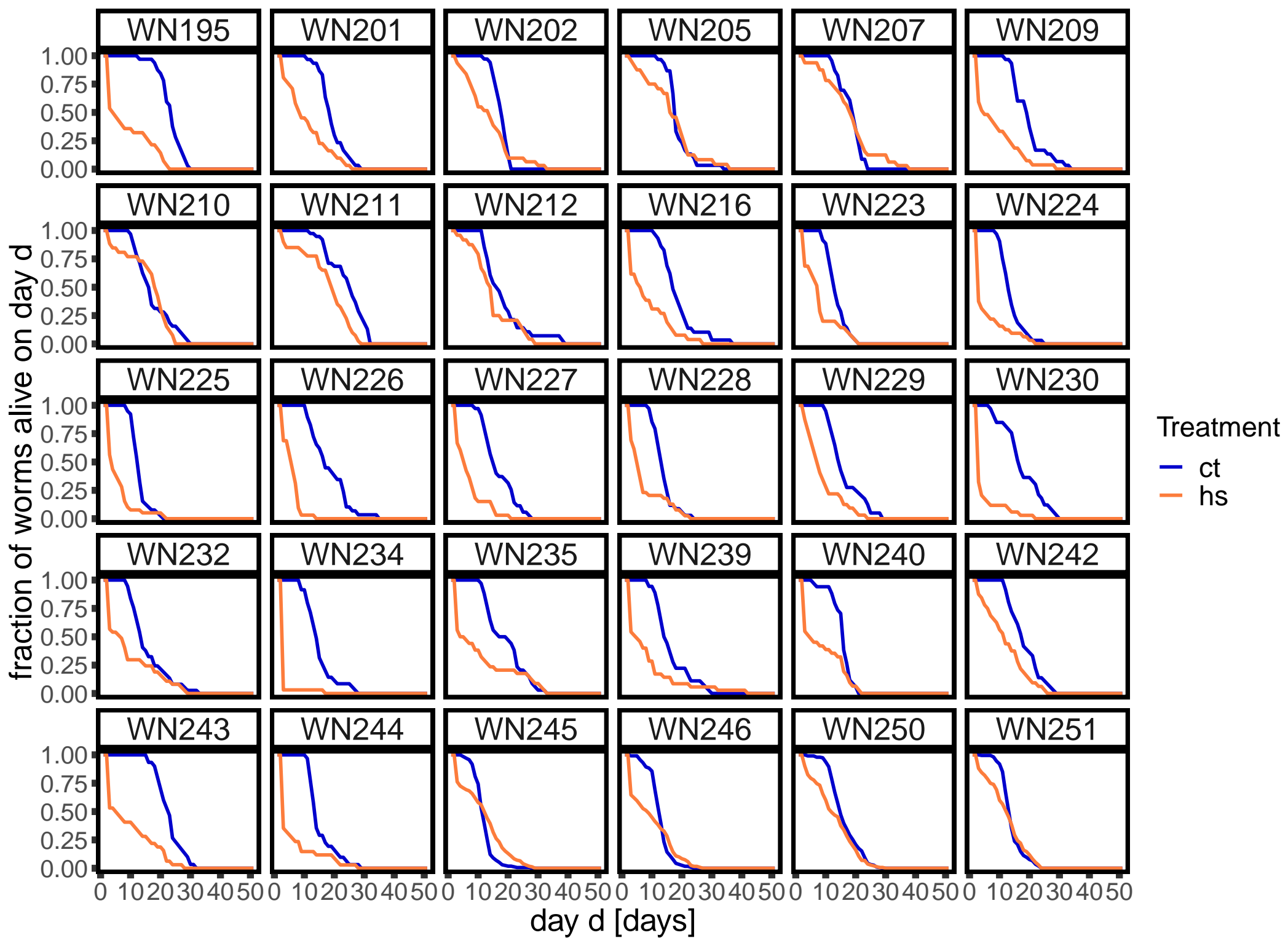

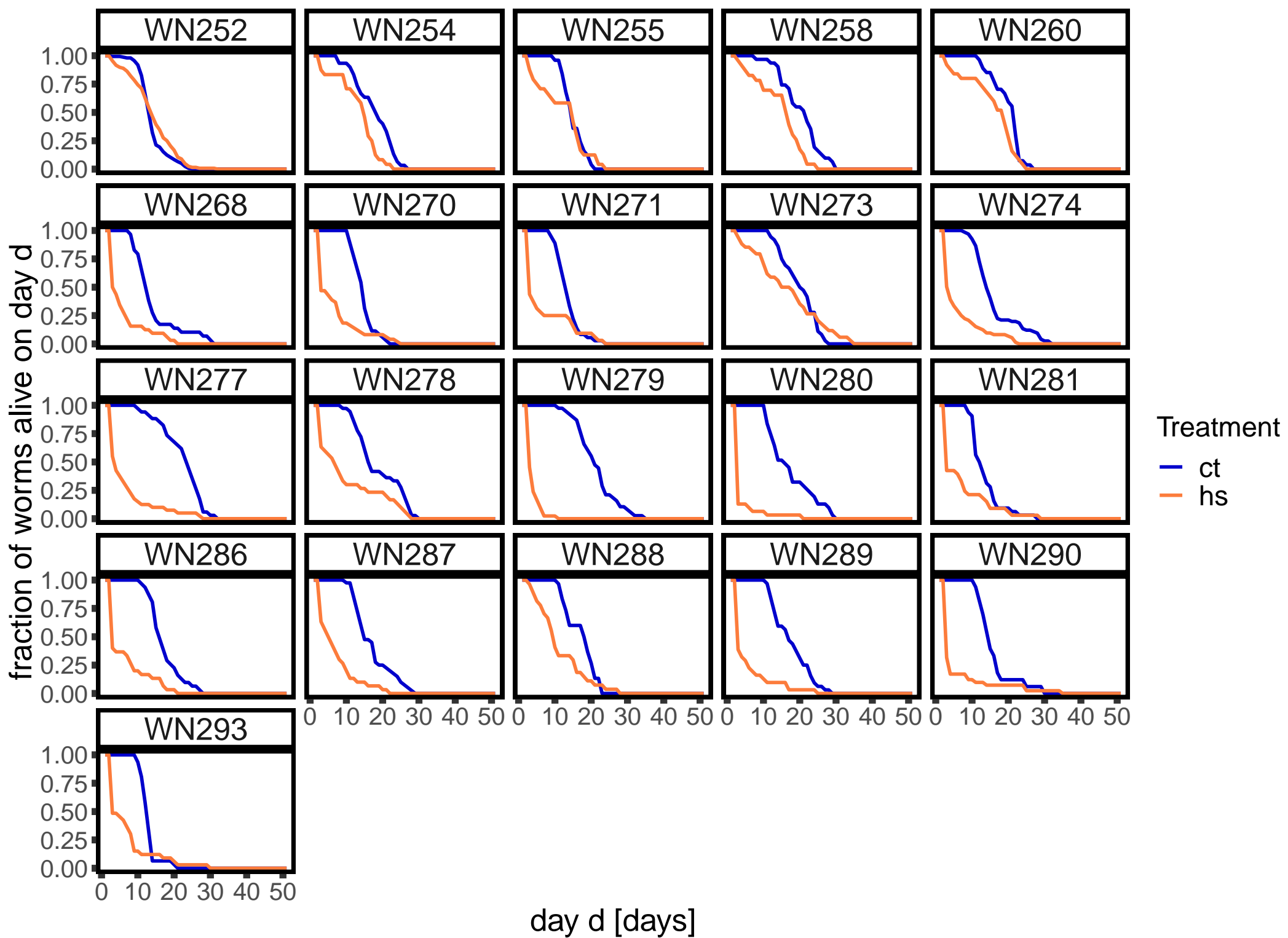

Supplement: Supplementary file 4 — Additional file 4. PDF file containing 85 figures (i.e. one figure for each genotype) depicting survival curves in heat-stress and control conditions. Associated statistics output can be found in Additional file 1: Table S6. [file 12915_2019_725_MOESM4_ESM.pdf]
